# Are we prepared? The development of performance indicators for public health emergency preparedness using a modified Delphi approach

**DOI:** 10.1371/journal.pone.0226489

**Published:** 2019-12-23

**Authors:** Yasmin Khan, Adalsteinn D. Brown, Anna R. Gagliardi, Tracey O’Sullivan, Sara Lacarte, Bonnie Henry, Brian Schwartz

**Affiliations:** 1 Public Health Ontario, Toronto, Ontario, Canada; 2 Department of Medicine, Division of Emergency Medicine, University of Toronto, Toronto, Ontario, Canada; 3 University Health Network, Toronto, Ontario, Canada; 4 Dalla Lana School of Public Health, University of Toronto, Toronto, Ontario, Canada; 5 Faculty of Health Sciences, University of Ottawa, Ottawa, Ontario, Canada; 6 Office of the Provincial Health Officer, Ministry of Health, Government of British Columbia, Victoria, British Columbia, Canada; Iran University of Medical Sciences, ISLAMIC REPUBLIC OF IRAN

## Abstract

**Background:**

Disasters and emergencies from infectious diseases, extreme weather and anthropogenic events are increasingly common. While risks vary for different communities, disaster and emergency preparedness is recognized as essential for all nation-states. Evidence to inform measurement of preparedness is lacking. The objective of this study was to identify and define a set of public health emergency preparedness (PHEP) indicators to advance performance measurement for local/regional public health agencies.

**Methods:**

A three-round modified Delphi technique was employed to develop indicators for PHEP. The study was conducted in Canada with a national panel of 33 experts and completed in 2018. A list of indicators was derived from the literature. Indicators were rated by importance and actionability until achieving consensus.

**Results:**

The scoping review resulted in 62 indicators being included for rating by the panel. Panel feedback provided refinements to indicators and suggestions for new indicators. In total, 76 indicators were proposed for rating across all three rounds; of these, 67 were considered to be important and actionable PHEP indicators.

**Conclusions:**

This study developed an indicator set of 67 PHEP indicators, aligned with a PHEP framework for resilience. The 67 indicators represent important and actionable dimensions of PHEP practice in Canada that can be used by local/regional public health agencies and validated in other jurisdictions to assess readiness and measure improvement in their critical role of protecting community health.

## Introduction

The global experience with recent public health emergencies such as outbreaks of Ebola Virus Disease and differential impacts of climate change has public health workers and the general public asking: Are we prepared? The burden of morbidity and mortality from emergencies and disasters can be severe, resulting in public health systems investing substantial time and resources toward preparedness [1]. The public health system is the lead in responding to outbreaks and in minimizing the impact of diverse emergencies on health [2,3]. Public health sector activities in infectious disease emergencies include leading other emergency management organizations during outbreaks, conducting surveillance and investigation, implementing control measures, developing guidance for health-care practitioners, and communicating risks [3]. In addition, public health is the lead sector in preparing for the population health effects of non-infectious events incited by natural or anthropogenic hazards. Emergency preparedness levels have been a concern globally in past emergencies; for example, Canada’s response to the 2003 Severe Acute Respiratory Syndrome outbreak raised a number of issues: lack of surge capacity in the clinical and public health systems, difficulties with timely access to laboratory testing and results, and weak links between public health and the health care system were clear gaps in preparedness [3].

Recognizing complex and system-level challenges that affect emergency preparedness efforts globally, the World Health Organization (WHO) has called for all countries to create resilient integrated systems that can be responsive and proactive to any future threat, although this remains a knowledge gap [4,5]. While risks vary for different communities, disaster and emergency preparedness is recognized as essential for all nation-states [4,6]. Local and regional public health agencies aim to mitigate risks and protect population health; however, they face challenges to ensure readiness for potential emergencies ranging widely in likelihood and impact. Further, investments change over time with economic and policy priorities, which can influence the resources available for this purpose. Thus, the ability to define and measure essential elements of public health emergency preparedness (PHEP) is important for local and/or regional public health agencies.

Measurement and reporting of performance indicators has been shown to impact system performance [7]. In Canada, the Canadian Institutes for Health Information and Statistics Canada report indicators of health status and health care system performance [8]; in addition, performance measurement has been used in Canada to inform health system decision-making [9]. The precise ways measurement and reporting influence health systems, however, remains unclear [10]. In recent years, increasing attention has been paid to performance measurement for the public health system [11,12]. While preparedness metrics are few in the literature [13–15], the pressure for public health agencies to articulate their degree of preparedness is increasing. Globally, countries are asked to meet targets aimed at reducing disaster risks in their communities, which includes health impacts [6], and the International Health Regulations (IHR) require that all nations report on indicators aligned with the IHR [16,17]. As nation-states examine their own readiness, indicators for relevant jurisdictional levels have been developed by some countries. For example, the United States (US) has examined aspects of preparedness in the context of national health security and emergency planning [18,19], including the concept of resilience [20,21], but measurement considering resilience relevant to and actionable for practice in local/regional public health is lacking.

Approaches in PHEP include event or risk-based planning, such as planning for the health impacts of an international sporting event, and all-hazards planning which aims to achieve preparedness for a range of possible hazards, both infectious (i.e. influenza) and non-infectious (i.e. natural disasters). The all-hazards approach is viewed as essential for public health system-level readiness, enabling effective and efficient preparedness that accounts for the difficulty in predicting the type and severity of events [14,22,23]. The conventional cycle of emergency management includes four phases which are (1) prevention/mitigation, (2) preparedness, (3) response and (4) recovery; public health agency activities relate to all four phases [24]. In this study, we focus on preparedness as upstream activities and actions that promote enhanced public health system capacity and resilience throughout all four phases. It is important to note that in Canada, PHEP addresses population-level preparedness, distinct from clinical care and health care facility preparedness. Communication and integration of preparedness activities between sectors like health care, government and the community is, however, often a responsibility of public health agencies. Relevant levels of the public health system in Canada are local or regional (varies by province/territory), provincial/territorial, and federal. We consider all three as the public health system, and we identify local/regional public health agencies as the primary locus of public health service delivery in Canada [3,25].

Defining a PHEP framework, establishing indicators, measuring performance, and supporting quality improvement (QI) can be viewed in a continuum to support building system resilience. Conceptual frameworks or maps serve as a starting point for performance measurement and QI [7]. “Indicators only indicate” and will never entirely capture the complexity of a system, making clarity and conceptualization about what the system is aiming to do essential [7]. To address the important task of ensuring readiness and creating resilient systems, our previous work developed a framework which identifies the essential elements of PHEP relevant to Canada, and considers the complexity of the public health system and emergency context [26]. The framework for resilience includes eleven essential elements and constitutes an evidence-based approach to defining PHEP for local/regional public health agencies and supporting practice for community health protection from disaster risks. In developing the framework, we noted that promoting resilience for public health systems requires consideration of complex aspects of preparedness such as social infrastructure [26,27]; for example, assessment of workforce capacity is influenced by individual workers’ willingness to respond [28]. In addition, addressing challenges across these systems may require measuring dimensions such as network strength or “connectivity” of relevant stakeholders [29]. The framework for resilience thus conceptualizes the essential elements to consider in measuring PHEP. The objective of this study is to identify and define a set of PHEP indicators aligned with the framework to advance performance measurement for local/regional public health agencies.

## Methods

### Approach

The modified Delphi method is an iterative survey and consultative process useful for indicator development in health research for fields with a limited evidence base like PHEP indicators [30,31]. We used a modified Delphi technique with two rounds of online surveys based on a scoping review and indicators suggested by the panel (second round only) [31]. The use of existing literature to inform the first round is an established modification to the Delphi which enhances the efficiency of a time-consuming open-ended question only round [31]. Reporting details according to standards for Delphi studies are found in S1 Table [30]. The study used an Integrated Knowledge Translation (iKT) approach and a steering committee of knowledge users, defined as professionals likely to use the results, that was consulted at key milestones [32]. Research ethics approval was obtained from Public Health Ontario and University of Ottawa Ethics Review Boards.

### Panel selection

This national study was conducted in Canada, where health services and programs are provided at the provincial/territorial level for ten provinces and three territories. In Canada, regional health authorities or networks generally include more than one municipality, while locally-organized health services are based at the municipal level [33]. Leaders involved in PHEP in Canada include local public health officials, provincial public health and health emergency management partners, and federal public health and health system partners.

Purposive sampling augmented by snowball recruitment was employed to deliberately select PHEP experts for a national sample of public health leaders and decision-makers [34]. Rationale for the sample definition is to ensure that key indicators in PHEP were identified by individuals with knowledge and experience specifically in PHEP, and who hold leadership roles and/or have clear responsibility for PHEP within their health unit, agency or jurisdiction, and for whom indicators would be relevant [31]. Medical Officers of Health (MOHs), Associate MOHs, Environmental Health Officers, and other leaders or decision-makers with experience and/or expertise in PHEP from the federal, provincial and municipal levels were recruited. We aimed to identify 20–30 PHEP experts across Canada and establish a heterogeneous composition of the panel [31,35]. In the performance measurement indicator literature, selection of expert participants is described through a process of nomination, which we employed to recruit established experts in PHEP [36]. A nomination process by email was thus used to identify experts in the field of PHEP based on experience, scholarship or reputation in their organization or jurisdiction [31,36].

The nomination process resulted in 48 PHEP nominees. Thirty-eight nominees were invited to participate based on geographic and professional diversity. Five nominees declined the invitation due to availability. Consistent with the criteria for nominations, the final Delphi panel was comprised of 33 experts representing senior-level positions spanning all jurisdictional levels across 12 of 13 provinces and territories. Self-reported areas of expertise included public health preparedness, response and management (63.6%) and health services emergency preparedness, response and management (57.6%). Other key areas of expertise included communicable diseases (42.4%) and environmental health (39.4%). The majority of the panel (78.7%) had over ten years of experience, with 42.4% of the panel with 20+ years of experience in their field. A profile of the expert panel characteristics is found in Table 1.

**Table 1:** Characteristics of public health emergency preparedness expert panel members.

Characteristics	All Members, No. (%) (n = 33)
**Current Position**	
Chief Medical Officer of Health/Deputy Health Officer	5 (15.2)
Medical Health Officer/Associate Medical Health Officer	6 (18.2)
Public Health Emergency Management Unit/ Program Leader	5 (15.2)
Health Emergency Management Unit/Program Leader	8 (24.2)
Operations Leader	2 (6.1)
Environmental Health Unit/Program Leader	1 (3.0)
Other key decision maker	6 (18.2)
**Organization**	
Local public health agency/regional health authority	7 (21.2)
Provincial/territorial public health organization/agency	10 (30.3)
Provincial/territorial government organization/agency	9 (27.3)
Federal government organization/agency	3 (9.1)
First Nations health authority	1 (3.0)
Other organizational category	3 (9.1)
**Jurisdictional Level**	
Local/municipal/regional jurisdictional level	6 (18.2)
Provincial/territorial jurisdictional level	22 (66.7)
Federal jurisdictional level	3 (9.1)
Other jurisdictional category	2 (6.1)
**Province/Territory of Employment**	
Ontario^a^	6 (18.2)
British Columbia	5 (15.2)
Alberta	3 (9.1)
Manitoba	3 (9.1)
Northwest Territories	3 (9.1)
Nova Scotia	3 (9.1)
Quebec	3 (9.1)
Newfoundland and Labrador	2 (6.1)
Saskatchewan	2 (6.1)
New Brunswick	1 (3.0)
Nunavut	1 (3.0)
Prince Edward Island	1 (3.0)
**Area(s) of Expertise**^**b**^	
Public health emergency preparedness and response/ Public health emergency management	21 (63.6)
Health emergency preparedness and response/ Emergency management	19 (57.6)
Communicable diseases	14 (42.4)
Environmental health	13 (39.4)
Research scholarship	3 (9.1)
Other area of expertise	4 (12.1)
**Years of Experience**	
5–9 years	7 (21.2)
10–14 years	8 (24.2)
15–19 years	4 (12.1)
20+ years	14 (42.4)

^a^ Includes representatives from the federal jurisdictional level.

^b^ Total is greater than 100% because participants may have more than one area of expertise.

### Data collection and analysis

A scoping review was used to identify and extract existing indicators for PHEP from the literature [37]. A librarian-assisted search strategy was developed and four databases were explored for relevant, English language, peer-reviewed literature. Grey literature searches included web searches, government research reports and key documents collected from knowledge users. The search strategy and related keywords for peer reviewed and grey literature is found in S1 Appendix Tables 1 and 2, respectively. The Preferred Reporting Items for Systematic Reviews and Meta-analysis (PRISMA) was used to map the number of records identified, included and excluded and the reasons for exclusion. The study selection process was followed by data extraction and data charting according to the descriptive numerical summary approach and conducted by two team members. Quality appraisal was conducted using the Meta Quality Appraisal Tool which is a tool specific to public health research [38]. The tool was used to qualitatively appraise the strengths and weaknesses of included studies by assessing relevancy, reliability, validity, and applicability to public health. Grades of high, moderate or low were assigned based on qualitative assessment of these dimensions, with a focus on validity of the development process for existing indicators, including description of the methodology used, and were reported in the data charting table.

The data from the final group of articles were synthesized with a hybrid approach of deductive and inductive thematic analysis, using NVivo 10. Themes were identified from extracted indicators, corresponding with each framework element. Extracted indicators corresponding to the PHEP framework were assessed for relevance to local/regional public health agency practice. Themes were used by the research team to develop and refine lists of indicators for inclusion in the round one survey by framework element.

Panel members were asked to rate each indicator based on criteria for quality indicators [7]. The United Kingdom’s National Health Service Institute for Innovation and Improvement has established a systematic approach to developing indicators using criteria of importance, validity, possibility, meaning and implications. The knowledge user steering committee provided feedback on quality indicator criteria and the criteria of importance and actionability were most relevant for the early stage of indicator development and included for indicator rating. Importance and actionability were defined respectively as: (1) this indicator is a key priority in public health preparedness for emergencies; and (2) this indicator is under the control of the local or regional public health agency. The survey asked participants to rate each indicator on both criteria on a seven-point Likert scale. Open-ended questions augmented the round one survey to elicit suggestions for additional indicators and obtain feedback on indicator clarity. The round 1 survey was input to the web-based platform Acuity4. The survey was piloted with experts who were not panel members but met criteria as a PHEP expert. Piloting aimed to assess clarity of the data collection instrument, functionality of the online format, and relevance of companion documents. Survey administration was managed by a research coordinator; participants were emailed a personalized URL and a companion document explaining the PHEP framework and indicator extraction/development. Three weekly attempts were made to contact non-respondents [31].

Responses were exported to Microsoft Excel for analysis. Ratings of agreement (5–7) and disagreement (1–4) were calculated into a percentage reflecting the level of panel consensus for each criterion statement by indicator. An *a priori* cut-off for consensus of 70% was used based on published ranges [31]. Indicators that achieved 70% consensus as both important and actionable were retained as PHEP indicators after round 1. Indicators that reached consensus as both *not* important and *not* actionable (disagreement consensus of 70%) were discarded. Finally, indicators that achieved 70% consensus on importance or actionable but not both were deemed as *unclear* and were retained for revision according to panel feedback. Sensitivity analyses were carried out to examine the thresholds for consensus [31,36]. New indicators suggested by the panel during round 1 were extracted and analyzed using thematic analysis as there may be multiple descriptors of the same indicator [39]. First, multiple reviews of the raw data were conducted. Second, manual coding was completed and a set of unique themes (i.e. indicators) produced. Based on the resultant themes, a group of new indicators were developed for rating in round two.

The round two survey included revised versions of the indicators with unclear consensus for re-rating and the new suggested indicators. A summary of panel feedback and results of round one accompanied the round two survey link. Open-ended questions enabled participants to comment on the indicators. Consensus level of agreement was analyzed based on round two responses. Indicators from round two rating were retained, discarded or deemed to have unclear consensus. The third round was a meeting of the panel, with both web-conferencing and in-person participation. A summary of round two panel feedback was distributed in advance. Indicators with unclear consensus were revised and discussed to achieve final consensus to retain or discard. Anonymous rating was conducted using the polling feature in Adobe Connect to achieve final consensus for retaining or discarding indicators. The meeting was audio-recorded and transcribed to document panel feedback.

In keeping with the iKT approach in this study, the steering committee was consulted at key milestones. These included development of indicators from the scoping review; survey piloting; interpretation of survey results; and review and feedback on the final indicator list.

## Results

### Search results

The librarian-assisted search yielded 4,516 articles and 117 grey literature sources. After screening, a total of six peer-reviewed articles and thirteen grey literature sources were included in the final group for indicator extraction. The flow of selection is outlined by a PRISMA diagram in S1 Appendix Fig 1. The data charting table, descriptive summary, and quality assessments are found in S2 Table. From the literature, 397 indicators spanning 62 themes were extracted and classified by the 11 PHEP framework elements [26]. Themes and indicators extracted from the literature relevant to PHEP are summarized in Table 2. Based on the themes, 62 indicators were identified for round one panel rating.

**Table 2:** Public health emergency preparedness framework and indicator themes.

PHEP Framework Element	Theme	No. of Indicators
Governance and leadership	1. Systems and structures (including Incident Command System)	10
	2. Managing uncertainty and decision-making	3
	3. Leadership, roles and responsibilities	10
	4. Policy, protocol, standards, legal requirements	34
Planning process	1. Possession and maintenance of a written all-hazards response plan	3
	2. Continuity of operations plan	1
	3. Recovery plan	1
	4. Long-term emergency planning	2
	5. Relevance to local risks	7
	6. State-wide disaster planning	1
	7. Plans exercised and assessed regularly	6
	8. Planning for strategic national stockpile material	3
Practice and experience	1. Evaluate and practice plans (e.g., drills and exercises)	13
	2. Capability development (e.g., following plans, developing skills, etc.)	6
	3. Educational sessions to increase awareness	1
	4. Activation (e.g., Incident Management System, plans, exercises)	3
	5. Planning of the exercises	4
	6. Flexibility or adaptability	1
Risk analysis	1. Defining/understanding risks (e.g., assessments of safety, health, etc.)	8
	2. Reporting risk	2
	3. Hazard identification and risk assessment	8
	4. Informing response	2
	5. Data accessibility	3
	6. Control measures (e.g., mitigating hazard impacts)	4
	7. Needs assessment	2
	8. Vulnerability assessment (e.g., populations)	1
Learning and evaluation	1. Post-incident evaluation	8
	2. Quality improvement through exercises and responses and a comprehensive exercise plan	6
Resources	1. Equipment and supplies	6
	2. Emergency resource needs assessment	3
	3. Medical countermeasure dispensing	3
	4. Medical countermeasure receiving	6
	5. Pharmaceutical shortages	2
	6. Human resources	7
	7. Infrastructure and logistics	5
	8. Emergency funding	3
	9. Equipment and supplies	6
Workforce capacity	1. Training	20
	2. Surge capacity and available staff	17
	3. Staff awareness of plans, roles, responsibilities	3
	4. Knowledge and skills	4
	5. Staff safety and personal protective equipment	3
	6. Volunteer management	5
	7. Human resources plan	3
Collaborative networks	1. Stakeholder engagement	14
	2. Mutual aid agreements, procedures, plans	17
	3. Emergency operations coordination	4
	4. Determine roles/responsibilities, coordinate services	9
	5. Connectivity	4
Community engagement	1. Raising awareness of risks in the community	3
	2. Community involvement in policies and processes	3
	3. Awareness of high-risk populations	4
	4. Public trust	6
Communication	1. Risk communication systems (e.g., policy, guidance, and mechanisms)	2
	2. Internal and partner communication and coordination	2
	3. Communication approach	1
	4. Communication plans for staff, public, media	9
Surveillance and monitoring	1. Health surveillance and epidemiological investigation	22
	2. Biological monitoring and laboratory testing	14
	3. Environmental health monitoring	12
	4. Monitor, analyze and recommend mitigation activities	4
	5. Thresholds for implementing enhanced surveillance in the community	18

### Modified Delphi

Three rounds of data collection occurred between November 2017 and January 2018. The response rate for round one was 100%. Of the 62 indicators proposed for rating, 41 achieved consensus agreement of 70% on importance and actionability and were retained after the first round. The remaining 21 indicators had unclear consensus. Nineteen indicators achieved consensus on importance but not actionability. Two indicators reached consensus on actionability but not importance. Comments pertaining to actionability generally related to jurisdictional responsibility, and/or resource/financial constraints out of local/regional level control. The results of round one by indicator are provided in S2 Appendix Tables 1 and 2. Indicators with unclear consensus were revised; however, indicators were not modified to address actionability comments if the indicator reached consensus for importance. Panel suggestions resulted in an additional 14 new indicators. A list of indicators suggested by the panel is found in S2 Appendix Table 3. A total of 35 indicators were incorporated into the round 2 survey.

Round one achieved a 100% response rate. Of the 35 indicators, 23 reached the 70% level of consensus on both importance and actionability; the remaining 12 indicators had unclear consensus (S2 Appendix Tables 4 and 5). Feedback on the 12 indicators was reviewed and indicators revised accordingly, with the 12 indicators forming the basis for discussion at the final meeting.

**Fig 1:**
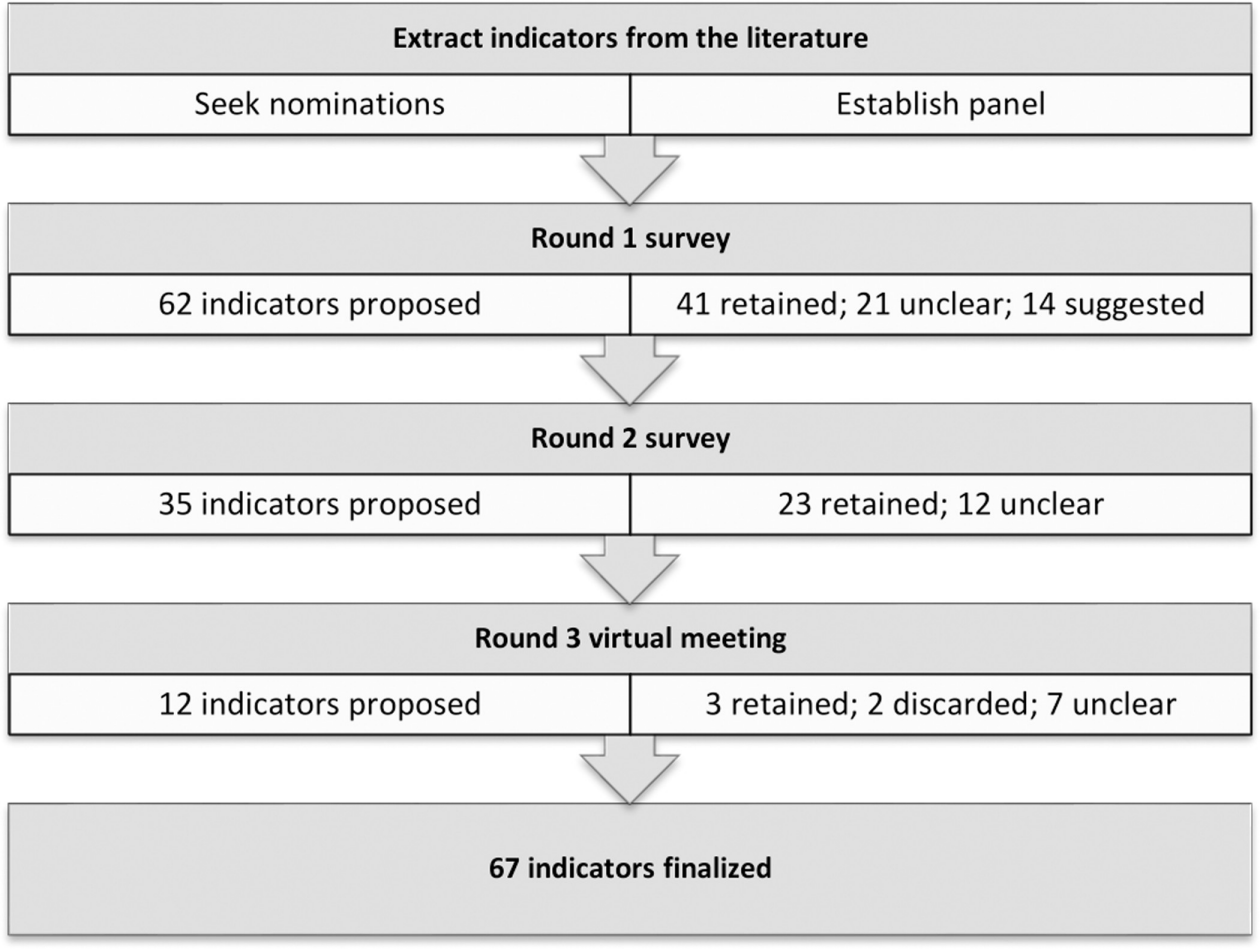
Modified Delphi process used to select indicators for public health emergency preparedness.

During the course of the half-day round three meeting, participation ranged from 22–28 members (67–85%). Analyses of indicator ratings were adjusted according to the number of votes received in each poll. At the meeting, three indicators reached consensus and two indicators were discarded (S2 Appendix Tables 6 and 7). Seven indicators were deemed to be important but not actionable (S2 Appendix Table 8). Summary qualitative comments from round 3 are provided in S2 Appendix Table 9. Fig 1 outlines the modified Delphi process used to identify PHEP indicators relevant to local/regional public health agencies. The results of the analyses of the final set of indicators are presented in Table 3.

**Table 3:** Final set of public health emergency preparedness indicators.

	Important			Actionable		
Indicators (N = 67)	Median (IQR)	No.	Consensus Level (%)	Median (IQR)	No.	Consensus Level (%)
**Governance and Leadership (12 indicators)**						
1. The public health agency is a member of a local/regional structure for health-sector emergency management that aims to coordinate health system preparedness for emergencies. Network partners involved in this structure may include, for example, acute care, primary care, or emergency medical services, depending on the jurisdiction.	7 (1)	32	97	6 (2)	29	87.9
2. The public health agency’s policies describe the authority and procedures under which it would respond to an emergency as the lead agency.	6 (1)	32	97	6 (2)	28	84.8
3. The public health agency’s policies define the conditions and procedures for using incident management structures and processes to coordinate agency activities in emergencies.	6 (1)	32	97	6 (2)	27	81.8
4. The public health agency aligns its emergency plans and/or protocols with provincial, territorial and/or federal policy on public health and emergency management.	6 (1)	31	93.9	6 (1)	31	93.9
5. The public health agency’s policies describe the authority and procedures under which it would respond to an emergency in a supportive role to the lead agency.	6 (1)	31	93.9	6 (0)	29	87.9
6. The public health agency’s policies define the conditions and procedures for escalating response to an emergency, including processes for declaring an event multi-jurisdictional.	6 (1)	31	93.9	6 (1)	25	75.8
7. The public health agency is a member of a local/regional multidisciplinary structure that aims to reduce community risks to emergencies and disasters. Network partners involved in this structure may include transportation, planners, industry, local/regional elected officials.	6 (1)	31	93.9	5 (2)	24	72.7
8. The public health agency’s policies align with requirements for reporting to the provincial/territorial and/or federal public health authority on community health risks in the context of an emergency; for example, radio-nuclear, chemical or biosecurity events.	6 (2)	31	93.9	5 (1)	28	84.8
9. The public health agency engages with policy-makers to address gaps in policy and/or legislation that pertain to the effectiveness of its emergency management plans and/or protocols.	5 (1)	24	92.3	5 (1)	22	91.7
10. The public health agency’s policies define processes for establishing a clear leader in the context of emergency.	7 (1)	30	90.9	6 (2)	26	78.8
11. The public health agencies plans are linked to the mandate of network partners in vertical or horizontal multi-jurisdictional response to emergencies; for example, responsibilities for different levels of government.	5 (0)	23	88.5	5 (1)	23	88.5
12. The public health agency has defined leadership competencies for individuals that may act as agency leaders in an emergency. These may include: established effective relationships, local knowledge, credible, flexible, trusted, ethical.	6 (1)	28	84.8	6 (1)	25	75.8
**Planning Process (6 indicators)**						
13. The public health agency reviews its emergency plans and/or protocols with involved departments and/or programs internal to the agency.	6 (1)	33	100	6 (1)	33	100
14. The roles and responsibilities of the public health agency for responding to all-hazards emergencies are defined in agency plans and/or protocols.	7 (1)	31	93.9	6 (1)	30	90.9
15. The roles and responsibilities for the public health agency in ensuring business continuity during an emergency are established in agency plans and/or protocols.	6 (1)	31	93.9	6 (1)	31	93.9
16. The public health agency has a process to support priority-setting decisions in the allocation of limited resources in the context of emergencies.	6 (1)	29	87.9	6 (3)	24	72.7
17. The public health agency’s emergency management plans and/or protocols relate to all phases of a disaster (i.e. Prevention/mitigation, preparedness, response, and recovery).	6 (1)	28	84.8	6 (2)	28	84.8
18. Linkages between the public health agency and network partners’ emergency plans and/or protocols are discussed with involved network partners.	6 (1)	28	84.8	6 (2)	24	72.7
**Risk Assessment (5 indicators)**						
19. The public health agency uses the results of the risk assessment to inform relevant plans/protocols for emergency management, business continuity and/or risk reduction.	6 (1)	32	97	6 (1)	30	90.9
20. The public health agency’s risk assessment process includes an analysis of organizational capacity to manage the identified risks.	6 (1)	32	97	6 (1)	29	87.9
21. The public health agency uses locally relevant data to inform risk assessment. Examples of data sources may include: communicable diseases, vector-borne diseases, food and water testing, population health determinants, non-communicable diseases such as injuries.	6 (1)	31	93.9	6 (1)	27	81.8
22. The public health agency conducts a comprehensive risk assessment for all-hazards emergencies at regular intervals (e.g. annually, or when a new threat is identified) to adapt to emerging risks.	6 (2)	28	84.8	6 (1)	27	81.8
23. The public health agency’s risk assessment process considers the preparedness capacity of populations that may be at increased risk in the context of emergencies.	6 (2)	28	84.8	6 (2)	24	72.7
**Resources (6 indicators)**						
24. The public health agency has established procedures to facilitate timely dispensing of physical resources to the community in the context of emergencies (e.g., may include medical prophylaxis and/or treatment).	6 (1)	32	97	6 (2)	29	87.9
25. The public health agency has or has access to a dedicated emergency preparedness coordinator, or similar position, led by an individual experienced in emergency management.	7 (1)	32	97	5 (1)	26	78.8
26. The public health agency has mechanisms to secure or reallocate financial resources to support response to and recovery from an emergency.	5 (1)	26	96.3	4 (1)	20	80
27. The public health agency has or has access to a system to support management of physical resources relevant to emergencies; for example, equipment, supplies or medical prophylaxis and/or treatment (e.g. may include tracking, monitoring and/or reporting components).	6 (1)	31	93.9	5 (1)	25	75.8
28. The public health agency is familiar with established procedures for the exceptional procurement of physical resources relevant to the emergency context, including procedures for procurement outside of business hours; for example, equipment, supplies or medical prophylaxis and/or treatment from the provincial, territorial or federal government.	6 (1)	30	90.9	6 (1)	25	75.8
29. The public health agency has dedicated financial resources to support planning and preparedness activities for emergencies.	7 (2)	28	84.8	5 (2)	24	72.7
**Collaborative Networks (4 indicators)**						
30. The public health agency has mechanisms for contacting network partners in the event of an emergency.	7 (1)	33	100	6 (1)	30	90.9
31. The public health agency has demonstrated the ability to perform cooperative activities with network partners. This ability may be demonstrated, for instance, during real or simulated emergencies.	6 (1)	32	97	6 (1)	27	81.8
32. The public health agency has partnerships and/or mechanisms to access specialized expertise relevant to community risks; for example, environmental health, biosecurity, toxicology, transportation companies, legal advice	6 (1)	32	97	6 (2)	24	72.7
33. The public health agency has mutual aid agreements in place with health-sector network partners that describe how resources and/or services will be shared during an emergency, including meeting demands for surge capacity.	6 (2)	31	93.9	6 (2)	24	72.7
**Community Engagement (4 indicators)**						
34. The public health agency provides and/or endorses education programs directed at the public to raise awareness about preparedness for relevant community risks.	6 (1)	30	90.9	5 (1)	28	84.8
35. The public health agency dedicates time for the continuous development of relationships with community organizations relevant to preparedness for local risks and the agency context; for example, building relationships with members of the public and/or advocacy groups that represent the public.	6 (1)	27	81.8	6 (1)	28	84.8
36. The public health agency has or participates in an established structure to facilitate inclusion of community considerations in relevant aspects of public health emergency management. For example, a community advisory committee to inform emergency mitigation, planning and/or recovery including members of the public and/or advocacy groups that represent the public.	5 (1)	27	81.8	5 (1)	27	81.8
37. The public health agency and/or its network partners engage with Indigenous communities regarding emergencies and related risks. Engagement may include community-specific risk assessments, plans and/or protocols, and inclusion of Indigenous knowledge where possible and appropriate.	6 (1)	27	81.8	5 (3)	24	72.7
**Communication (11 indicators)**						
38. The public health agency has a mechanism to formally or informally coordinate joint messaging with relevant network partners in a timely manner.	6 (1)	33	100	6 (1)	27	81.8
39. The public health agency has structures to ensure message consistency with network partners; for example, regular network partner coordination meetings, incident management systems.	7 (1)	33	100	5 (2)	24	72.7
40. The public health agency has capacity for redundancy in communication platforms in the context of an emergency; for example, using alternate platforms in power outages or if regular communication channels are down.	6 (1)	32	97	5 (1)	26	78.8
41. The public health agency communication strategy uses multiple communication platforms to facilitate timely information-sharing in the context of an emergency; for example, town-hall meetings, websites, social media, spokespersons, information call lines/centres.	7 (1)	31	93.9	6 (1)	32	97
42. The public health agency has identified trained spokesperson(s) for the agency relevant to community risks and the emergency context.	6 (1)	31	93.9	6 (1)	30	90.9
43. The public health agency has access to communications personnel that are dedicated to the emergency and appropriately trained in crisis communication.	6 (1)	31	93.9	5 (1)	28	84.8
44. The public health agency has a process for monitoring the media, including social media, to rapidly identify rumours and correct misinformation.	6 (1)	31	93.9	6 (1)	26	78.8
45. The public health agency communication strategy includes plans and/or procedures for ensuring cultural competency and/or sensitivity to impacted communities for relevant risks and the emergency context. This includes procedures for translation of messages to relevant languages.	7 (1)	30	90.9	6 (2)	29	87.9
46. The public health agency has developed communication strategies for multiple audiences in advance of emergencies, based on its risk assessment.	6 (2)	30	90.9	6 (1)	29	87.9
47. The public health agency has a process for the public and media to ask questions and voice concerns; for example, town hall meetings, social media, information call lines/centres.	7 (1)	30	90.9	6 (1)	29	87.9
48. The public health agency communication strategy includes procedures for directly reaching citizens during an emergency, if required. For example, door-to-door, giving out pamphlets, engaging in informal street/neighbourhood gatherings.	6 (2)	27	81.8	6 (1)	26	78.8
**Workforce Capacity (7 indicators)**						
49. The public health agency has a roster of its workforce available for the management of, or potential for, emergencies on a 24/7/365 basis.	7 (1)	32	97	6 (1)	31	93.9
50. The public health agency has established policies and procedures for supporting staff during an emergency with respect to their health and wellbeing; for example, on personal safety, mental wellbeing, family commitments.	7 (1)	30	90.9	6 (2)	29	87.9
51. The public health agency has a structure and/or mechanism to support multi-disciplinary emergency management relevant to community risks; for example, a multi-disciplinary team of public health professionals, epidemiologists, and environmental health officers.	7 (1)	30	90.9	6 (1)	27	81.8
52. The public health agency has a workforce professional development plan for training its staff that is specific to emergency management topics; for example, content of emergency plans/protocols, incident management systems, communications.	6 (1)	30	90.9	6 (1)	25	75.8
53. The public health agency workforce has demonstrated the ability to perform cooperative activities as an organization in the context of emergencies. This may be demonstrated, for instance, during exercises or activations.	6 (1)	30	90.9	5 (1)	25	75.8
54. The public health agency has an up to date inventory of staff trained in emergency management topics; for example, content of emergency plans/protocols, incident management systems, communications.	6 (2)	27	81.8	6 (1)	28	84.8
55. The public health agency conducts needs assessments regularly to determine the emergency management training needs of its workers.	6 (2)	26	78.8	5 (2)	24	72.7
**Surveillance and Monitoring (4 indicators)**						
56. The public health agency has the capability for or access to enhanced and/or event-based surveillance systems relevant to local/regional risks.	7 (1)	33	100	6 (3)	24	72.7
57. The public health agency has protocols and/or processes for information-sharing with network partners for purposes of surveillance of relevant risks; for example, with agricultural, veterinary or environmental surveillance systems.	6 (1)	31	93.9	5 (1)	26	78.8
58. The public health agency uses a syndromic surveillance and/or other early warning systems to detect potential public health emergencies in a timely manner.	6 (1)	30	90.9	5 (3)	24	72.7
59. The public health agency has the capability to conduct rapid health risks and/or needs assessments for communities recently impacted by emergencies.	6 (2)	29	87.9	5 (2)	24	72.7
**Learning and Evaluation (3 indicators)**						
60. The public health agency applies a self-assessment process to emergency management. This process may be applied to tests, exercises, simulations and/or emergency plan activations and agency responses.	6 (1)	28	84.8	6 (2)	28	84.8
61. The public health agency self-assessment process is used to identify capabilities, strengths and/or assets to describe successes relevant to emergency management.	6 (2)	28	84.8	6 (2)	27	81.8
62. The public health agency self-assessment process is used to inform improvement actions; for example, identifying responsible groups for corrective actions and establishing timelines for change.	6 (1)	28	84.8	6 (2)	26	78.8
**Practice and Experience (5 indicators)**						
63. The public health agency practices its plans and/or protocols that are relevant to emergency management; for example, the agency emergency response plan, the business continuity plan. Practice may include table tops, exercises, simulations, or activations for emergencies.	7 (1)	31	93.9	6 (2)	28	84.8
64. The public health agency conducts regular needs assessments to determine the needs for organizational practice of emergency plans and/or protocols; for example, the emergency response plan, the business continuity plan. The assessment may consider recent table tops, exercises, simulations, or activations in response to emergencies.	6 (2)	31	93.9	6 (1)	27	81.8
65. Public health agency management and staff have demonstrated the ability to adjust plans and/or protocols for emergencies in the context of new knowledge, uncertain science, and/or differences in professional opinions. This ability may be demonstrated during real or simulated emergencies.	6 (2)	31	93.9	6 (1)	25	75.8
66. The public health agency has sufficient resources to practice plans and/or protocols relevant to emergency management; for example, the emergency response plan, the business continuity plan. Practice may include table tops, exercises, or simulations.	7 (1)	29	87.9	6 (1)	25	75.8
67. Public health agency practice of emergency management activities (e.g., table tops, exercises, simulations) includes the regular attendance of both management and staff.	6 (2)	28	84.8	6 (2)	27	81.8

Over the three rounds of the survey, indicators were confirmed or identified for all domains of the PHEP framework. There was, however, a range in number of indicators identified per element, with Governance and Leadership having the most indicators identified at 12, followed by Communication with 11. Learning and Evaluation had the fewest at three; Surveillance and Monitoring, Collaborative Networks, and Community Engagement had four. The number of indicators per other element ranged from five to seven. In total, 76 indicators were proposed for rating across all three rounds; of these, 67 were considered to be important and actionable PHEP indicators.

## Discussion

The objective of our study was to identify and define a set of indicators to advance PHEP performance measurement and guide quality improvement for local/regional public health agencies. A total of 67 indicators were developed and categorized according to an empirically-derived PHEP framework. This development of indicators by a locally-based, nationally representative expert panel represents a potentially valuable contribution to evidence-informed public health practice with particular relevance to local/regional public health.

PHEP indicator sets have previously been developed for various jurisdictions. Generally these have been oriented around accountability for funding and resource allocation for preparedness [18]. However, recent research on resilient health systems indicates that funding accountability-focused metrics may not capture a meaningful conceptualization of PHEP to answer the question ‘Are we prepared?’, when it comes to protecting community health [20,21]. Further, while improved preparedness has been demonstrated in organizations with experience managing a disaster [40], indicators and greater and more consistent measurement can enhance learning and improvement after real or simulated events. Continuous QI is an important part of public health practice and an emphasis on learning is a cornerstone for resilience-oriented approaches [4,6]. This study advances the PHEP measurement literature in that it aligns with existing targets and regulations, but furthers it through the lens of tools to support monitoring, learning and improvement.

Some local/regional public health agency PHEP indicator sets use existing datasets [18]. Although this has benefits for feasibility in creating snapshots of preparedness, it poses challenges for QI. For example, the indicators may not be part of a model anchored around the agency as the focus and thus may not be specific to this context. Further, indicators may not be aligned with activities within agency jurisdiction and control. Our set of indicators aligns with a PHEP framework comprised of essential elements identified based on empiric data for local/regional public health agencies [26]. The indicators correspond with the essential elements and were assessed through this study as relevant to PHEP, achieving high consensus agreement and consistency for importance. Our list of indicators contributes to the applied public health literature in that they represent actionable aspects of PHEP practice for public health agencies. While specific to this context, our work contributes to global efforts to gauge preparedness given the indicators were derived based on existing global indicators, such as the Joint External Evaluation tool [17].

There are limitations to this study. Like much indicator development, the evidence underlying metrics is limited and largely reliant on grey literature. There were few examples in the literature of rigorously derived and validated indicators. Given the broad scope of PHEP, our literature review may not have been exhaustive. This was mitigated by conducting an in-depth search of peer-reviewed and grey literature, contacting experts requesting documents, and examining key websites in the field. Indeed, new knowledge emerged as our study was in progress. Specifically, the European Centers for Disease Control (ECDC) released a report describing PHEP core competencies for European Union member states in 2017 [41]. While a new approach for the ECDC, this work was an adaptation of a US-based model published previously [15,26,41], and the indicators corresponding with the model were derived from similar documents [17,27]. Our indicator development process used a breadth of sources and aligned with an empirically-developed conceptual framework. Further, the panelists evaluated each of the proposed indicators and had the opportunity to suggest additional ones. Future work will benefit from validation of these indicators in practice.

Our study results have implications for policy and practice. Public health agencies can establish and use these indicators to create a baseline and measure PHEP. While the final list confirmed 67 important and actionable indicators, another seven indicators were found to be important but *not* actionable. This additional group of indicators is highly relevant to PHEP practice due to the high importance ratings; however, these seven indicators highlight the complexity around measuring PHEP and the PHEP system. For example, a Governance and Leadership indicator: Provincial/territorial authorities and local/regional public health agencies jointly develop policies and/or structures defining the agency mandate in public health emergency management met consensus at 88.9% for importance but only 50% for actionability. The “joint” aspect of this indicator was identified as key to its importance; however, it may not be actionable based on the context of a single agency and may be most useful for local public health agencies as they assess the collective readiness of their region, advocate and plan to increase readiness.

The indicators are many and varied, which may raise concerns about the feasibility of QI and burden of reporting. While challenging, this reflects the diversity of risks, actors and organizations with which emergency preparedness planners engage. The range for the number of indicators by element was likely influenced by the literature as there were more existing indicators for concepts such as governance, communication and resources, while other concepts such as collaboration and learning were less explored. It is important to note, however, that in keeping with a complex system, the elements are seen as interconnected and adaptive. For example, aspects of collaboration are captured through other elements, including Governance and Leadership, Planning Process and Communication.

Future research should address the usefulness of these indicators in practice. It will be important to assess gaps in indicators that relate to key elements of the PHEP framework. Further, some indicators–around communication and community engagement in particular–require multiple perspectives for validation. Research should be directed toward developing standardized tools for measurement that are relevant across organizations. Another approach uses a logic model or strategy map where lead indicators or those likely to change earlier (often process indicators) can be related against lag indicators or those that are likely to change later (often outcome indicators). Our framework suggests that success across all elements is likely necessary for successful response to disasters and emergencies [26], making examination of correlations between elements or indicators challenging. To further advance the science of performance measurement for PHEP, field-based piloting and validation of the indicators will be beneficial.

## Implications

1. This study presents relevant and useful indicators for local/regional public health agencies to assess practice in PHEP and guide improvement.
2. This study addresses a knowledge gap in the literature in developing an indicator set specific to local/regional public health agencies that considers the complexity of the PHEP context and emergencies.
3. The indicators are situated in a framework that includes empirically-derived essential elements for PHEP for local/regional public health agencies, relevant governance structures and forums, and ethics and values as principles.
4. Given the ability of emergencies to spread beyond jurisdictional boundaries, it will be important to have national and continued global approaches to PHEP measurement.
5. These indicators can be used for assessment and for quality improvement purposes.

## Conclusions

In conclusion, this study adds to the evidence base of PHEP by developing a suite of indicators aligned with a PHEP framework for resilience. The indicator set was derived by employing a three-round modified Delphi survey using a national expert panel in Canada. The rigour and transparency of our process is a novel contribution to the PHEP literature and may assist other countries in considering how to transfer the findings to their context. The 67 indicators represent important and actionable dimensions of PHEP practice that can be used and validated by local/regional public health agencies to assess readiness and measure improvement in their critical role of protecting community health.

## Supporting information

S1 Table Guidance on conducting and reporting Delphi studies (CREDES) criteria. (DOCX)

S1 Appendix Scoping review search. (DOCX)

S2 Table Scoping review data charting. (DOCX)

S2 Appendix Indicators. (DOCX)

## References

[1] WHO Ebola Response Team. Ebola Virus Disease in West Africa—The First 9 Months of the Epidemic and Forward Projections. N Engl J Med. 2014;371: 1481–1495. 10.1056/NEJMoa1411100 25244186 PMC4235004

[2] Costich JF, Scutchfield FD. Public health preparedness and response capacity inventory validity study. J Public Health Manag Pract. 2004;10: 225–233. 10.1097/00124784-200405000-00006 15253518

[3] Public Health Agency of Canada (PHAC). Learning from SARS: Renewal of public health in Canada | Leçons de la crise du SRAS: Renouvellement de la santé publique au Canada. 2003;0-662-34984-9.: 1–22.

[4] World Health Organization. A strategic framework for emergency preparedness. 2017. Available from: http://apps.who.int/iris/bitstream/handle/10665/254883/9789241511827-eng.pdf

[5] Rutter H, Savona N, Glonti K, Bibby J, Cummins S, Finegood DT, et al The need for a complex systems model of evidence for public health. Lancet. 2017;390: 2602–2604. 10.1016/S0140-6736(17)31267-9 28622953

[6] United Nations Office for Disaster Risk Reduction (UNISDR). Sendai framework for disaster risk reduction 2015–2030. 2015. Available from: http://www.unisdr.org/files/43291_sendaiframeworkfordrren.pdf

[7] NHS Institute for Innovation and Improvement and the Association of Public Health Observatories. The good indicators guide: understanding how to use and choose indicators. June 1, 2007.

[8] Canadian Institute for Health Information (CIHI). Health Indicators 2013. 2013.

[9] Veillard J, Huynh T, Ardal S, Kadandale S, Klazinga NS, Brown AD. Making health system performance measurement useful to policy makers: aligning strategies, measurement and local health system accountability in Ontario. Healthcare Policy. 2010;5: 49–65. 21286268 PMC2831733

[10] Levesque JF, Sutherland K. What role does performance information play in securing improvement in healthcare? A conceptual framework for levers of change. BMJ Open. 2017;7. 10.1136/bmjopen-2016-014825 PMC5724223 28851769

[11] National Association of County and City Health Officials (NACCHO). National Public Health Performance Standards (NPHPS). 2013.

[12] Corso L, Lenaway D, Beitsch L, Landrum L, Deutsch H. National public health performance standards: driving quality improvement in public health systems. J Public Health Manag Pract. 2010;16: 19–23. 10.1097/PHH.0b013e3181c02800 20009640

[13] McCabe OL, Barnett DJ, Taylor HG, Links JM. Ready, willing, and able: a framework for improving the public health emergency preparedness system. Disaster Med Public. 2010;4: 161–168. 10.1001/dmp-v4n2-hcn10003 20526139

[14] Nelson C, Lurie N, Wasserman J. Assessing public health emergency preparedness: concepts, tools, and challenges. Annu Rev Public Health. 2007;28: 1–18. 10.1146/annurev.publhealth.28.021406.144054 17129174

[15] Stoto M. Measuring and assessing public health emergency preparedness. J Public Health Manag Pract. 2013;19: S16–21. 10.1097/PHH.0b013e318294b0e3 23903388

[16] World Health Organization. IHR core capacity monitoring framework: checklist and indicators for monitoring progress in the development of IHR core capacities in state parties. 2013. Available from: https://www.who.int/ihr/Processes_of_IHR_Monitoring_framework_and_Indicators.pdf?ua=1

[17] World Health Organization. Joint external evaluation tool: International Health Regulations (2005). 2016. Available from: https://apps.who.int/iris/bitstream/handle/10665/204368/9789241510172_eng.pdf;jsessionid=8107D04E9BB61D4C9F4D19F2C08061E9?sequence=1

[18] National Health Security Preparedness Index (NHSPI). Explore the index release. 2018. Available from: https://nhspi.org/

[19] National Association of County and City Health Officials (NACCHO). Project public health ready criteria: version 8.1. 2016.

[20] Toner E, Shearer M, Sell T, et al., Centers for Disease Control and Prevention, John Hopkins Center for Health Security. Health sector resilience checklist for high-consequence infectious diseases-informed by the domestic US Ebola response. 2017. Available from: http://www.centerforhealthsecurity.org/our-work/pubs_archive/pubs-pdfs/2017/HCID_Final_Report_05.23.2017.pdf

[21] Toner ES, McGinty M, Schoch-Spana M, Rose DA, Watson M, Echols E, et al A community checklist for health sector resilience informed by Hurricane Sandy. Health Secur. 2017;15: 53–69. 10.1089/hs.2016.0079 28192055 PMC5551499

[22] Marcozzi DE, Lurie N. Measuring healthcare preparedness: an all-hazards approach. Isr J Health Policy Res. 2012;1: 42 10.1186/2045-4015-1-42 23098101 PMC3502095

[23] Adini B, Goldberg A, Cohen R, Laor D, Bar-Dayan Y. Evidence-based support for the all-hazards approach to emergency preparedness. Isr J Health Policy Res. 2012;1: 40–46. 10.1186/2045-4015-1-40 23098065 PMC3494498

[24] Emergency Management Policy Directorate, Public Safety Canada. An Emergency Management Framework for Canada, Second Edition. 2011.

[25] Public Health Agency of Canada. Lessons Learned Review: Public Health Agency of Canada and Health Canada Response to the 2009 H1N1 Pandemic. 2010.

[26] Khan Y, O'Sullivan T, Brown AD, Tracey S, Gibson J, Généreux M, et al Public health emergency preparedness: A framework to promote resilience. BMC Public Health. 2018;18. 10.1186/s12889-018-6250-7 PMC6280369 30518348

[27] World Health Organization Regional Office for Europe. Strengthening health system emergency preparedness: toolkit for assessing health system capacity for crisis management. 2012. Available from: http://www.euro.who.int/__data/assets/pdf_file/0008/157886/e96187.pdf

[28] Barnett DJ, Balicer RD, Thompson CB, Storey JD, Omer SB, Semon NL, et al Assessment of local public health workers' willingness to respond to pandemic influenza through application of the extended parallel process model. PLoS ONE. 2009;4: e6365 10.1371/journal.pone.0006365 19629188 PMC2711331

[29] Dorn BC, Savoia E, Testa MA, Stoto MA, Marcus LJ. Development of a survey instrument to measure connectivity to evaluate national public health preparedness and response performance. Public Health Rep. 2007;122: 329–338. 10.1177/003335490712200306 17518304 PMC1847495

[30] Jünger S, Payne SA, Brine J, Radbruch L, Brearley SG. Guidance on Conducting and REporting DElphi Studies (CREDES) in palliative care: recommendations based on a methodological systematic review. Palliat Med. 2017;31: 684–706. 10.1177/0269216317690685 28190381

[31] Keeney S, Hasson F, McKenna H. Delphi technique in nursing and health research. First Edition ed. Ames, Iowa: Wiley-Blackwell; 2011.

[32] Canadian Institutes of Health Research. Guide to knowledge translation planning at CIHR: integrated and end-of-grant approaches. 2012. Available from: http://cihr-irsc.gc.ca/e/documents/kt_lm_ktplan-en.pdf

[33] Statistics Canada. Health regions and peer groups. 2015.

[34] Quinn PM. Chapter 5. Designing qualitative studies In: Anonymous Qualitative research and evaluation methods. Thousand Oaks, CA: SAGE Publications; 2015 pp. 243.

[35] Mcilfatrick S, Keeney S. Identifying cancer nursing research priorities using the Delphi technique. Journal of Advanced Nursing. 2003;42: 629–636. 10.1046/j.1365-2648.2003.02666.x 12787236

[36] Gagliardi A, Simunovic M, Langer B, Stern H, Brown AD. Development of quality indicators for colorectal cancer surgery, using a 3-step modified Delphi approach. Can J Surg. 2005;48: 441–452. 16417050 PMC3211732

[37] Levac D, Colquhoun H, O'Brien KK. Scoping studies: advancing the methodology. Implement Sci. 2010;5: 69 10.1186/1748-5908-5-69 20854677 PMC2954944

[38] Rosella L, Bowman C, Pach B, Morgan S, Fitzpatrick T, Goel V. The development and validation of a meta-tool for quality appraisal of public health evidence: Meta Quality Appraisal Tool (MetaQAT). Public Health. 2016;136: 57–65. 10.1016/j.puhe.2015.10.027 26993202

[39] Guest G, MacQueen K, Namey E. Applied Thematic Analysis. First ed. Thousand Oaks, CA: Sage; 2012.

[40] Seyedin H, Zaboli R, Ravaghi H. Major incident experience and preparedness in a developing country. Disaster Med Public. 2013;7: 313–318. 10.1001/dmp.2012.34 22851618

[41] European Centre for Disease Prevention and Control. Public health emergency preparedness—core competencies for EU member states. 2017. Available from: https://ecdc.europa.eu/sites/portal/files/documents/public-health-emergency-preparedness-core-competencies-eu-member-states.pdf

