# Supplementary material for: Are we prepared? The development of performance indicators for public health emergency preparedness using a modified Delphi approach
Source: PLoS One. 2019 Dec 23;14(12):e0226489. doi: 10.1371/journal.pone.0226489 (PMC6927653; doi:10.1371/journal.pone.0226489)
Supplement: S1 Appendix — (DOCX) [file pone.0226489.s002.docx]

# S2 Appendix: Scoping review search

# S2 Table 1. Keyword and vocabulary chart for scoping review search (databases searched: MEDLINE, Embase, Environment Complete and Scopus)

|  | Concept 1:  Emergencies | Concept 2:  Preparedness | Concept 3:  Performance Indicators |
| --- | --- | --- | --- |
| MeSH | Avalanches/  Biohazard Release/  Bioterrorism/  Chemical Hazard Release/  Civil Defense/  Cyclonic Storms/  Disaster Medicine/  Disaster Planning/  Disaster Victims/  Disasters/  Earthquakes/  (Emergencies/ and (Public Health/ or Public Health Practice/ or Disease Outbreaks/))  Emergency Shelter/  Epidemics/  Extreme Cold/  Extreme Heat/  Floods/  Landslides/  Mass Casualty Incidents/  Pandemics/  Radioactive Hazard Release/  Relief Work/  Rescue Work/  Strategic Stockpile/  Terrorism/  Tidal Waves/  Tornadoes/  Tsunamis/  Volcanic Eruptions/ | Community Health Planning/  Community Health Services/og  Cooperative Behavior/  Disaster Planning/  Emergency Medical Services/og  Health Facility Planning/  Health Plan Implementation/  Health Planning Guidelines/  Health Planning/  Health Priorities/  Health Resources/  Health Systems Plans/  Hospital Planning/  Interinstitutional Relations/  Operations Research/  Organizational Case Studies/  Public Health Administration/  Public Health Practice/  Public Health/  Regional Health Planning/  "Resilience, Psychological"/  Security Measures/  State Health Plans/  State Medicine/  Surge Capacity/  Systems Analysis/ | Accreditation/  Benchmarking/  Disaster Planning/st  Efficiency, Organizational/  Efficiency/  "Evaluation Studies as Topic"/  "evaluation studies".pt.  Health Care Evaluation Mechanisms/  Management Audit/  "Outcome and Process Assessment (Health Care)"/  "Outcome Assessment (Health Care)"/  "Process Assessment (Health Care)"/  Program Evaluation/  Public Health Administration/st  Public Health Practice/st  Public Health/st  "Quality Assurance, Health Care"/  Quality Control/  "Quality Indicators, Health Care"/  "Quality of Health Care"/  Quality Improvement/  "Task Performance and Analysis"/  "Time and Motion Studies"/  Total Quality Management/ |
| Title key-words | "all hazard"  "all hazards"  avalanche*  ((biohazard* or chemical* or radioactiv* or hazard*) adj3 (release* or spill* or accident*))  bioterroris*  blizzard*  "brush fire*"  brushfire*  "bush fire*"  bushfire*  "civil defense"  "communit* preparedness"  "critical event*"  cyclone  cyclones  disaster*  earthquake*  ((emergencies or incident or incidents or epidemic* or pandemic*) adj2 (prepar* or plan or plans or plann* or manag* or resilien* or respon* or ready or readiness or recover*))  "emergency management"  "emergency plan*"  "emergency preparedness"  "emergency response"  "extreme cold"  "extreme heat"  "extreme weather*"  flood*  "forest fire*"  "health* preparedness"  "heat alert*"  "heat wave*"  hurricane*  landslide*  "mass casualt*"  "severe weather*"  storm  storms  "system* preparedness"  terroris*  "tidal wave*"  tornado*  tsunami*  typhoon*  volcano*  "wild fire*"  wildfire*  "wild land fire*"  "wildland fire*" | countermeasure*  manag*  plan  plann*  plans  prepar*  "public health"  readiness  ready  recover*  resilien*  respon* | accountab*  accredit*  apprais*  assess*  audit*  benchmark*  criteria  criterion  effectiveness  efficacy  evaluat*  improve*  improving  index  indexes  indicator*  instrument  instruments  inventories  inventory  "lessons learned"  measur*  measure  measureable  measures  metric  metrics  perform*  ((preparedness or response) adj3 (capacity or capacities or capability or capabilities))  (quality adj3 (assess* or improv* or manag* or control* or assur*))  registries  registry  "return* on investment*"  ROI  score  "score card*"  scorecard*  scores  scoring  standard  standards  target  targets  (value adj1 (money or dollar*)) |
| Title/ abstract key-words |  |  | "performance measur*"  "performance data"  "preparedness measure*"  "realist evaluation"  "after action report*"  "state of the nation*" |

# S2 Table 2. Search strategy for scoping review

| # | Searches |
| --- | --- |
| 1 | Avalanches/ or Biohazard Release/ or Bioterrorism/ or Chemical Hazard Release/ or Civil Defense/ or Cyclonic Storms/ or Disaster Medicine/ or Disaster Planning/ or Disaster Victims/ or Disasters/ or Earthquakes/ or (Emergencies/ and (Public Health/ or Public Health Practice/ or Disease Outbreaks/)) or Emergency Shelter/ or Epidemics/ or Extreme Cold/ or Extreme Heat/ or Floods/ or Landslides/ or Mass Casualty Incidents/ or Pandemics/ or Radioactive Hazard Release/ or Relief Work/ or Rescue Work/ or Strategic Stockpile/ or Terrorism/ or Tidal Waves/ or Tornadoes/ or Tsunamis/ or Volcanic Eruptions/ or ("emergency preparedness" or "emergency plan*" or "emergency management" or "emergency response" or "all hazard" or "all hazards" or avalanche* or ((biohazard* or chemical* or radioactiv* or hazard*) adj3 (release* or spill* or accident*)) or bioterroris* or blizzard* or brushfire* or "brush fire*" or bushfire* or "bush fire*" or "civil defense" or "critical event*" or cyclone or cyclones or disaster* or earthquake* or ((emergencies or incident or incidents or epidemic* or pandemic*) adj2 (prepar* or plan or plans or plann* or manag* or resilien* or respon* or ready or readiness or recover*)) or "extreme cold" or "extreme heat" or "extreme weather*" or flood* or "forest fire*" or "heat alert*" or "heat wave*" or hurricane* or landslide* or "mass casualt*" or "severe weather*" or storm or storms or terroris* or "tidal wave*" or tornado* or tsunami* or typhoon* or volcano* or wildfire* or "wild fire*" or "wild land fire*" or "wildland fire*" or "system* preparedness" or "communit* preparedness" or "health* preparedness").ti,kw,kf. |
| 2 | "Resilience, Psychological"/ or Community Health Planning/ or Community Health Services/og or Cooperative Behavior/ or Disaster Planning/ or Emergency Medical Services/og or Health Facility Planning/ or Health Plan Implementation/ or Health Planning Guidelines/ or Health Planning/ or Health Priorities/ or Health Resources/ or Health Systems Plans/ or Hospital Planning/ or Interinstitutional Relations/ or Operations Research/ or Organizational Case Studies/ or Public Health Administration/ or Regional Health Planning/ or State Health Plans/ or State Medicine/ or Surge Capacity/ or Systems Analysis/ or Security Measures/ or Public Health/ or Public Health Practice/ or (prepar* or plan or plans or plann* or manag* or resilien* or respon* or ready or readiness or recover* or countermeasure* or "public health").ti,kw,kf. |
| 3 | Benchmarking/ or "Evaluation Studies as Topic"/ or "Outcome and Process Assessment (Health Care)"/ or "Outcome Assessment (Health Care)"/ or "Process Assessment (Health Care)"/ or Accreditation/ or Health Care Evaluation Mechanisms/ or Program Evaluation/ or "Quality Assurance, Health Care"/ or Quality Control/ or Quality Improvement/ or "Quality Indicators, Health Care"/ or "Quality of Health Care"/ or Total Quality Management/ or Efficiency, Organizational/ or Efficiency/ or "Task Performance and Analysis"/ or "Time and Motion Studies"/ or Management Audit/ or "evaluation studies".pt. or Disaster Planning/st or Public Health Administration/st or Public Health Practice/st or Public Health/st or (evaluat* or assess* or perform* or measur* or improve* or improving or inventory or inventories or (quality adj3 (assess* or improv* or manag* or control* or assur*)) or accredit* or audit* or effectiveness or efficacy or "lessons learned" or apprais* or accountab* or "return* on investment*" or ROI or (value adj1 (money or dollar*)) or indicator* or score or scores or scoring or measure or measures or criteria or criterion or instrument or instruments or index or indexes or target or targets or "score card*" or scorecard* or "performance data" or metric or metrics or measureable or standard or standards or registry or registries or ((preparedness or response) adj3 (capacity or capacities or capability or capabilities)) or benchmark*).ti,kw,kf. or ("performance data" or "performance measur*" or "preparedness measure*" or "realist evaluation" or "after action report*" or "state of the nation*").ti,ab,kw,kf. |
| 4 | 1 and 2 and 3 |
| 5 | limit 4 to (english or french) |
| 6 | limit 5 to yr="2000 -Current" |
| 7 | remove duplicates from 6 |

**Google search engine queries used in search of grey literature:**

1. disaster OR emergency planning OR preparedness OR response OR readiness OR management OR resilience OR recovery evaluation OR assess OR performance OR measure OR quality indicator OR measure OR metric OR scorecard
2. all-hazard OR critical-incident OR critical-event OR mass-casualty planning OR preparedness OR response OR readiness OR management OR resilience OR recovery evaluation OR assess OR performance OR measure OR quality indicator OR measure OR metric OR scorecard
3. bioterrorism OR terrorism planning OR preparedness OR response OR readiness OR management OR resilience OR recovery evaluation OR assess OR performance OR measure OR quality indicator OR measure OR metric OR scorecard
4. community-preparedness OR health-preparedness OR system-preparedness planning OR preparedness OR response OR readiness OR management OR resilience OR recovery evaluation OR assess OR performance OR measure OR quality indicator OR measure OR metric OR scorecard
5. "extreme weather*" OR "severe weather" planning OR preparedness OR response OR readiness OR management OR resilience OR recovery evaluation OR assess OR performance OR measure OR quality indicator OR measure OR metric OR scorecard
6. epidemic OR pandemic planning OR preparedness OR response OR readiness OR management OR resilience OR recovery evaluation OR assess OR performance OR measure OR quality indicator OR measure OR metric OR scorecard

Note: Only the first 100 search results were reviewed.

**Sites/domains strategically searched:**

1. Public Safety Canada: *.publicsafety.gc.ca/*
2. Government of Canada – Get Prepared: *.getprepared.gc.ca/*
3. Public Health Agency of Canada – Emergency Preparedness and Response *.phac-aspc.gc.ca/ep-mu/*
4. Alberta Emergency Management Agency: *.aema.alberta.ca/*
5. Emergency Management BC: *.gov.bc.ca/*
6. Manitoba Emergency Measures Organization: *.manitobaemo.ca/*
7. New Brunswick Emergency Measures Organization: *.gnb.ca/*
8. Newfoundland and Labrador Fire and Emergency Services: *.ma.gov.nl.ca/*
9. Northwest Territories Emergency Management Organization: *.maca.gov.nt.ca/*
10. Nova Scotia Emergency Management Office: *.gov.ns.ca/emo/*
11. Nunavut Emergency Management: *.cgs.gov.nu.ca/*
12. Emergency Management Ontario: *.emergencymanagementontario.ca/*
13. Prince Edward Island Emergency Measures Organization: *.peipublicsafety.ca/*
14. Quebec – Ministère de la sécurité publique: *.securitepublique.gouv.qc.ca/*
15. Saskatchewan Emergency Management Organization: *.gr.gov.sk.ca/*
16. Yukon Emergency Measures Organization: *.gov.yk.ca/emo/*
17. BC Centre for Disease Control: *.bccdc.ca/*
18. Institut national de santé publique du Québec: *.inspq.qc.ca/*
19. Public Health Agency of Canada: *.phac-aspc.gc.ca/*
20. Centers for Disease Control and Prevention: *.cdc.gov/*
21. European Centre for Disease Prevention and Control: *.ecdc.europa.eu/*
22. World Health Organization: *.who.int/*
23. European Union: *.europa.eu/*
24. Government of Australia: *.gov.au/*
25. Government of the United Kingdom: *.gov.uk/*
26. United Nations: *.un.org/*
27. United States Government: *.gov/*
28. Government of Canada: *.gc.ca/*
29. Organisation for Economic Co-operation and Development: *.oecd-ilibrary.org/*
30. World Bank: *.worldbank.org/*

**List of websites strategically searched:**

1. Resource Guide for Disaster Medicine and Public Health <http://disasterlit.nlm.nih.gov/> Federal Emergency Management Agency <http://www.fema.gov/>
2. World Association for Disaster and Emergency Medicine <https://wadem.org/>
3. National Emergency Management Association <http://www.nemaweb.org/>
4. International Association of Emergency Managers <http://www.iaem.com/>
5. Australian Emergency Management Knowledge Hub <https://www.emknowledge.gov.au/>
6. Canadian Federal and Provincial Emergency Management Agencies <https://cse.google.com/cse/publicurl?cx=011843882881040462305:nh8ooaox6pu>

**S2 Fig 1. PRISMA diagram for scoping review**

Records excluded
(n =4,533)

Studies included in scoping review
(n = 19)

Full-text articles assessed for eligibility
(n = 262)

Records screened
(n = 4,792)

Records after duplicates removed
(n =4,792)

Additional records identified through other sources
(n = 159)

## Identification

## Eligibility

## Included

## Screening

Records identified through database searching
(n = 4,660)

Full-text sources excluded
(n = 241)

Type of content excluded:

- General emergency management
- General public health management
- Hospital/clinic/personal preparedness
- Unrelated to indicators, or no clear methods of evaluation
- Evaluations of surveillance systems
- Low resource settings
