# Supplementary material for: Are we prepared? The development of performance indicators for public health emergency preparedness using a modified Delphi approach
Source: PLoS One. 2019 Dec 23;14(12):e0226489. doi: 10.1371/journal.pone.0226489 (PMC6927653; doi:10.1371/journal.pone.0226489)
Supplement: S2 Table — (DOCX) [file pone.0226489.s003.docx]

**S3 Table. Scoping review data charting**

| **Author, Year** | **Title** | **Purpose/Aim** | **Country; Jurisdictional Level** | **Hazard** | **Method of Development** | **Indicator Themes** | **PHEP Elements** | **Ethics/Values** | **Results** | **Validation** | **Quality Appraisal** |
| --- | --- | --- | --- | --- | --- | --- | --- | --- | --- | --- | --- |
| Asch, 2005[1] | A review of instruments assessing public health preparedness. | To review instruments that assesses level of preparedness of state and local public health departments to respond to health threats such as bioterrorism. | USA; Local/Regional and State | Chemical, biological, radiological, nuclear, explosive (CBNRE) | A literature review was undertaken to evaluate and assess peer reviewed articles using the Essential Public Health Services framework. Articles were evaluated for 1) clarity of measurement parameters; 2) balance between structural and process measures; 3) evidence of effectiveness, and 4) specification of an accountable entity. Instruments include community preparedness, hospital preparedness, and state-level health emergency preparedness. | 1. Monitor health problems to identify and solve community health problems (e.g., disease reporting, syndromic surveillance).  2. Diagnose and investigate health problems and health hazards in the community (e.g., information system capacity).  3. Inform, educate, and empower people about  Health issues.  4. Mobilize community partnerships and action to identify and solve health problems.  5. Develop policies and plans that support individual and community health efforts.  6. Enforce laws and regulations that protect health and ensure safety.  7. Link people to needed personal health services and assure the provision of health care when otherwise available.  8. Assure a competent public and personal health care workforce.  9. Evaluate effectiveness, accessibility, and quality of personal and population-based health services.  10. Research for new insights and innovative solutions to health problems. | Planning process; Practice and experience; Learning and evaluation; Resources; Collaborative networks; Community engagement; Communication; Surveillance and monitoring. | Not discussed | Most measures relied on subjective or structural measures, were lacking in evidence for  measures assessed, or did not clearly define which agency was responsible for accomplishing specific tasks or functions. | Expert opinion | Moderate |
| Babaie, 2016[2] | Developing a performance assessment framework and indicators for communicable disease management in natural disasters. | This study aimed to develop a performance assessment framework and indicators for communicable disease management in natural-hazard induced disasters. | Iran; Local/Regional and State | Natural (NAT) | Four development stages were undertaken:  (1) development of framework & indicators by undertaking a systematic review (published in a previous paper); (2) conducting interviews and focus groups  (n= 22, experts from the Center for Disease Control of the Ministry of Health) to assess the appropriateness of indicators; (3) developing a 167 item questionnaire (n=49, experts) further refined into 118 items; and (4) using an expert panel (n=11) to finalize indicator assessment resulting in 40 indicators. The weighting of indicators (n=8, experts) followed an analytical hierarchy process using criteria assessed on 7-point Likert scales: clear, relevant, adequate, economic, and monitorable. | The Inputs, process, products, and outcomes (IPPO) framework includes:  **Inputs:**  Man power; Equipment and supplies; Availability of guidelines and job action sheets.  Infrastructure: Availability of Incident Command Structure; Existence of health related databases.  Developing Surveillance system (SS): Establishing of health relief posts.  Coordination: Regional, national, and international coordination rates.  Funding: Emergency funding rate.  **Process:**  RHNA: Timeliness and completeness of RHNA; Environmental Health;  Morbidity case finding and reporting; Disease confirmation.  Trend analysis: Trend analyzing and feedback provision; Legislation; Data Security; Risk Communication;  Participation rate of media in reporting;  Surveillance System: Simplicity and flexibility of SS; Rate of under surveillance diseases/syndromes; Sensitivity and positive predictive value; Capacity of SS for predicting size and intensity of emergencies. | All elements | Not discussed | The IPPO framework with 40 indicators was found to be appropriate for performance assessment. The framework consists of seven main functions (immunization coverage, CD control, SS, outbreak management, environmental health, incidence and prevalence rate, and risk communication) and forty indicators  for four categories (input, process, products, outcomes). | Content validity was assessed throughout the development process using CDC experts. | High |

| **Author, Year** | **Title** | **Purpose/Aim** | **Country; Jurisdictional Level** | **Hazard** | **Method of Development** | **Indicator Themes** | **PHEP Elements** | **Ethics/Values** | **Results** | **Validation** | **Quality Appraisal** |
| --- | --- | --- | --- | --- | --- | --- | --- | --- | --- | --- | --- |
| Barnett, 2009[3] | Assessment of local public health workers’ willingness to respond to pandemic influenza through application of the extended parallel process model (EPPM). | To examine the influences of perceived threat and efficacy on Local Public Health Workers response willingness to pandemic influenza. | USA; Local/Regional | CBRNE | •John Hopkins Public Health Infrastructure Response Survey Tool (JH~PHIRST) was distributed to health department workers in three states; a total of 1835 workers responded. The survey includes:  •Demographic information: gender, education, role in emergency response, responsibility of family member, age, professional classification, years in present organization, years in profession.  •Attitude/beliefs towards a public health emergency response scenario of influenza pandemic based on a 10 point Likert scale (1=strong agreement; 10=weak agreement) with an additional option of "don't know".  •Responses were categorized according to the EPPM reference categories: Low threat/high efficacy; high threat/low efficacy; high threat/high efficacy. | Attitudes and beliefs towards pandemic influenza emergency, including: willingness to respond; perceived knowledge; perceived skills; perceived personal safety at work; perceived ability of health department to provide timely information; perceived need for pre-event preparation and training. | Workforce capacity Practice and experience | Not discussed | The JH~PHIRST can be used to understand how public health workers’ individual degrees of perceived threat (concern) and perceived efficacy (confidence) influence their willingness to respond. Local public health departments can use JH~PHIRST to design, implement  and evaluate training programs focused on readiness and response efforts. | Witte's EPPM & John Hopkins Public Health Infrastructure Response Survey Tool were developed by other researchers and commonly used in research. The validation process was not explained. | Moderate |
| Dorn, 2007[4] | Development of a survey instrument to measure connectivity to evaluate national public health preparedness and response performance. | To develop a quantifiable measure to evaluate linkages among individuals, organizations, and systems using the construct of "connectivity" ("the social cohesion or solidarity of a group of individuals as measured by the pattern of network ties"). | USA; Local/Regional, State and Federal | All hazard | •Items in the Connectivity Measurement Tool (CMT) were generated from  six focus groups with first responders (number of participants not specified).  •After the focus group, a panel of experts from Harvard Centre for Public Health Preparedness designed instrument accordingly (number of experts not specified).  •The instrument was piloted with 187 participants after completing a leadership training session; respondents were from the federal, state, and local levels.  •The empirical structure of the scale was determined by principal components analysis; the internal consistency was determined by Cronbach's alpha; mean values were compared using t- tests; effect size was estimated. | System connectivity;  Organizational connectivity;  Coworker connectivity;  Individual connectivity. | Collaborative networks | Not discussed | The Connectivity Measurement Tool was found to be a reliable measure of connectivity with preliminary evidence of construct validity. The final tool consists of a 34 item questionnaire to measure linkages among individuals, organization and systems across public health. | Principal components analysis explained 70% of variance in the model; internal reliability was strong (Cronbach's alpha ranged from 0.90 - 0.94); discriminant validity was consistent with the factor structure. | High |
| Gebbie, 2006[5] | Role of exercises and drills in the evaluation of public health in emergency response. | The objective of the study was to develop defined criteria for the evaluation of agency performance. | USA; Local/Regional | All hazard | •Criteria were developed using expert opinion, types and levels of exercises were identified using a critical review of national standard language developed by Department of Homeland Security (DHS).  •Criteria for evaluating local public health agency exercises were identified using a two round Delphi study with 26 member expert panel.  •Experts were from state or local public health; DHS regions; national response agencies; public health professional organizations; public health disaster specialists/academia.  •Round 1 survey grouped 50 agency level criteria into 10 categories to be evaluated as to whether it should be retained, modified, or eliminated; panel members were asked to comment or suggest new criteria.  •Round 2 survey grouped 46 potential criteria into 9 categories (74% response rate). The panel was asked to comment on final set of criteria. | (1) Initial response command and control (includes  7 criteria);  (2) Communication (includes 8 criteria);  (3) Early recognition/surveillance/epidemiology  (includes 7 criteria);  (4) Sample testing (includes 5 criteria);  (5) Evidence management (includes 2 criteria);  (6) Mass prophylaxis/immunization/pharmaceutical stockpiles (includes 6 criteria);  (7) Mass-patient care (includes 6 criteria);  (8) Mass-fatality management (includes 2 criteria); (9) Environmental surety (includes 3 criteria).  The menu of criteria for evaluation of local public health emergency drills and exercises is found on pp.177-179.  Table 4 on pp.180-181 lists the relationship of universal task list items to exercise evaluation criteria. | Planning process; Practice and experience;  Risk analysis;  Learning and evaluation; Resources; Workforce capacity; Community engagement; Communication; Surveillance and monitoring. | Not discussed | The criteria can be used to measure performance and identify areas for improvement as part of ongoing training and evaluation plans for evaluation of local public health emergency drills and exercises. The criteria can provide the metric for the Universal Task List which is organized according to the four USA Homeland Security Missions: Prevent, Protect, Respond and Recover. | Content was validated by the use of expert participants in the research study (>80% consensus was  achieved for final set). | High |
| Harvard School of Public Health, Center for Public Health Preparedness, 2013[6] | Public health emergency preparedness exercise evaluation toolkit; Section 6: Emergency preparedness exercise evaluation database (create your own form). | Public health agencies can use the toolkit as a resource to improve how emergency preparedness exercises are evaluated. | USA; Local/regional | All hazard | The toolkit was developed using measures tested in 60 emergency preparedness exercises. The evaluation toolkit was pilot tested with 14 partnering organizations representing 10 public health agencies and four health care agencies from eight states across the US. Exercise planners were interviewed about their experience on the use of the toolkit, the generated evaluation tool, and the usefulness of the data being gathered for the development of the exercise after-action report.[7] | Only measures specific to public health emergency preparedness in the Canadian context were selected from the online platform; key themes include:  Activation/Mobilization of Continuity of Operations Plans (COOP); Activation/Mobilization of emergency operations plan  Activation/Mobilization of joint information center; Activation/Mobilization of mutual aid agreements;  Apprised of situation updates;  Assessment of agent/cause of the incident;  Assessment of incident location;  Assessment of material capabilities and needs for optimal response to the incident;  Assessment of personnel capabilities and needs for optimal response to the incident;  Assignment of leadership roles;  Communication among departments during downtime; Communication with vulnerable or at-risk populations; Coordination and integration of response partners to support the response;  Coordination of information sharing across agencies; Development and management of risk; Communication for internal staff;  Gathering and updating of accurate information; Horizontal dissemination of information messaging;  Identification of and strategies to recruit volunteers in a response; Identification of strategies to communicate with patient's families and the public;  Identify specific personnel and non-personnel resources needed to support response logistics; Management of volunteers needed for the response. | Governance and leadership;  Planning process;  Collaborative networks;  Surveillance and monitoring;  Resources;  Workforce capacity;  Communication;  Learning and evaluation. | Ethics and values were highlighted in the indicator themes of 1) Communication with vulnerable or at-risk populations, which included sub-indicators that discussed sharing information in languages and formats that match population demographics; reaching vulnerable populations; having well established relationships with community leaders and organizations to relay messages; and  2) Identification of strategies to and communicate with patient's families and the public. Sub-indicators included: outreach to appropriate community groups to reach vulnerable populations; strategies to communicate with patients’ families and the general public; and identifying the dominant language of patients. | Exercise planners found the tool appropriate for use in evaluation and data collected by the tool was useful in performance assessment.[7] | Content was validated by the pilot testing process with exercise planners. | Moderate |
| Lee, 2013[8] | Developing a tool to measure and compare organizations’ resilience. | To develop a benchmark resilience survey tool to assist organizations with assessing strengths and weaknesses to determine areas for improvement. | New Zealand; Local/Regional | All hazard | •Survey items were operationalized using a four-point Likert scale from strongly agree to strongly disagree.  •A pilot study was conducted to check usability of the tool and face validity of items - Senior managers and staff members from four organizations completed the survey and were interviewed for feedback.  •249 individuals from 68 organizations (including 13 industry sectors) completed an online version.  •Items include McManus' Relative Overall  Resilience Model and a new model proposed in the study.  •Principal axis factor analysis was employed and reliability was tested using Cronbach’s alpha. | Silo mentality;  Capability and capacity of internal resources;  Staff engagement and involvement;  Information and knowledge;  Leadership, management, and governance structures;  Innovation and creativity;  Devolved and responsive decision making;  Internal and external situation monitoring and reporting;  Overall adaptive capacity;  Planning strategies; Participation in exercises;  Proactive posture;  Capability and capacity of external resources;  Recovery priorities;  Overall planning. | All elements | Ethics and values were briefly mentioned when discussing the importance of mission statements of organizations. | Factor analysis revealed that a two factor solution of adaptive capacity and planning was found to be the best fit.  Internal reliability was excellent (0.950); alpha scores for individual indicators ranged from 0.677-0.945. The final benchmark resilience survey tool is comprised of planning and adaptive capacity, and is measured using 13 indicators (53 items). | Construct validity, concurrent validity and reliability testing. | High |
| Lis, 2017[9] | How to choose? Using the Delphi method to develop consensus triggers and indicators for disaster response. | To identify key decisions along the continuum of care (conventional, contingency, and crisis) and the critical triggers and data elements used to inform those decisions concerning public health and health care response during an emergency. | USA; Local/Regional and State | All hazard | •The classic Delphi technique was used with clinicians to gather regional level triggers and indicators; followed by a modified Delphi approach using a combined workshop and single round survey with panelists from public health and health coalitions to identify state-level triggers and indicators.  •17/19 participants completed first two rounds of the clinical survey and 14/17 panelists completed the last round;  •18 participants completed the state-level  survey;  •A 5 point Likert scale was used to rate  importance;  •Consensus was set at 70% | Regional level indicators: 1. Resource conservation;  2. Medical staff; 3. Changing standards of care; 4. Liability protection of providers; 5. Pharmaceutical shortages; 6. Increased surveillance; 7. Health care capacity issues; 8. Standardized infection control; 9. Standardized regional health care practice guidelines; 10. Health care mutual aid.  Indicators and Triggers- State-level Survey:  1. Changing standards of care; 2. Resource conservation; 3. Pharmaceutical shortages; 4. State guidance for resource prioritization; 5. State guidance for health care operations; 6. State guidance for Infection control; 7. State guidance for Clinical practice/treatment; 8. Requesting interstate/international aid -Staff; 9. Requesting interstate/international aid -Stuff; 10. Requesting federal resource - Staff; 11. Requesting federal resource - Stuff; 12. Authorities of the Secretary of Health - Isolation and quarantine; 13. Authorities of the Secretary of Health - Social distancing; 14. Authorities of the State Secretary of Health - Adapting national guidance. | Governance and leadership; Collaborative networks;  Risk analysis; Surveillance and monitoring; Resources; Workforce capacity. | Not discussed | 122/213 triggers and indicators reached consensus as important by clinical panelists and 110/140 triggers and indicators were identified as important by PHEP  and coalition leads.  122 clinical and 110 state-level triggers and indicators were identified to support decision-making for  health care and public health during a disaster. | Content validity was established by the use of experts in the Delphi process. | High |

| **Author, Year Title Purpose/Aim Country; Hazard Method of Development Indicator Themes PHEP Elements Ethics/Values Results Validation Quality Appraisal**  **Jurisdictional Level** | | | | | | | | | | | |
| --- | --- | --- | --- | --- | --- | --- | --- | --- | --- | --- | --- |
| Mann, 2004[10] | Public health preparedness for mass-casualty events: A 2002 state by state assessment. | To characterize state-level disaster readiness and correlate readiness with existing programs providing an organized response  to medical emergencies. | USA; Local/Regional and State | All hazard | Summary information was drawn from the disaster preparedness component of a standardized trauma needs assessment developed by the Trauma-Emergency Management Systems Program within the Health Resources and Services Administration; survey consisted  of 27 items administered in 50 states to assess disaster readiness; a state panel with representatives from public health, EMS, and trauma systems completed the survey. Items inquired whether statewide coverage was available for each attribute of readiness (yes/no), if no, respondents were asked to estimate the proportion of the state covered by existing programs. An overall readiness score was calculated for each state by summing percentage points for readiness attributes with state-wide coverage (100%) or partial coverage (80%) for all 27 items; the overall score was correlated with the number of funded programs per state. | State-wide disaster planning; coordination; training; resource capacity; preparedness for biological/chemical terrorism. | Planning process; Collaborative networks; Practice and experience; Resources; Communication; Surveillance and monitoring; Governance and leadership. | Not discussed | The survey was developed by the US department of Health and Human Services, Health Resources and Services Administration. The measures were developed by a panel of experts. No further details of how the measures were developed were reported. The survey results provide a baseline which states can target funding  and determine the progress needed towards enhanced disaster preparedness. | Content of the survey was validated by public health and health care professionals. Details regarding development are not explained in the article. | Low |
| Centers for Disease Control and Prevention,  2011[11] | Public Health Preparedness Capabilities: National Standards for State and Local Planning | The capabilities assist state and local planners in identifying gaps in preparedness, determining  the specific jurisdictional priorities, and developing plans for building and sustaining capabilities. | USA; Local/Regional and State | All hazard | The content of each public health preparedness capability is based on evidence-informed documents, applicable preparedness literature, and subject matter expertise gathered from across the federal government and the state and  local practice community. In developing this document, CDC reviewed key legislative and executive directives to identify state and local public health preparedness priorities. These include the following: Pandemic and All-Hazards Preparedness Act (PAHPA), which authorizes state and local preparedness funding; U.S.A Department of Homeland Security (DHS) Homeland Security Presidential Directives 5, 8, and 21; and the National Health Security Strategy (NHSS). 200 subject matter experts were involved in weekly development meetings and stakeholder consultation groups. The methodology was peer reviewed by the Board of Scientific Counselors for CDC's Office of Public Health Preparedness and Response. | CDC identified 15 public health preparedness capabilities. Each capability includes a definition of the capability and list of the associated functions, performance measures, tasks, and resource considerations.  Bio-surveillance: public health laboratory testing; public health surveillance and information management.  Epidemiological investigation: community resilience; community preparedness; community recovery.  Countermeasures and mitigation: medical countermeasure dispensing; medical materiel management and distribution; non-pharmaceutical interventions; responder safety and health; incident Management.  Information management: emergency public information and warning.  Information sharing, surge management: fatality management; mass care; medical surge; volunteer management. | All elements | Not discussed | The 15 capability sections in this document were validated by subject matter experts. These capabilities are intended to serve as national standards that state and local public health departments can use to advance their preparedness planning. | Content was validated by engaging with stakeholder groups in the development process. No further validation or evaluation was discussed in this document. | Moderate |
| National Association of County and City Health Officials (NACCHO),  2016[12] | Project Public Health Ready  Criteria (Version 8.1) | Project Public Health Ready (PPHR) is a competency-based training and recognition program that assesses preparedness and assists local health departments (LHDs), or groups of LHDs collaborating as a region, to respond to emergencies. | USA; Local/Regional | All hazard | This is a collaborative project among NACCHO, CDC, and Columbia University’s Center for Health Policy. The project team consulted with an expert advisory committee during the development phases. In 2002, 13 LPHDs were selected to pilot test the draft PPHR certification requirements and provide feedback on their experience working with partners to implement the project at the local level. | Goal 1: written all-hazards plan;  Goal 2: training assessment and workforce development plan;  Goal 3: demonstration of readiness through exercise or real response. | All elements | Community preparedness involves collaboration with community stakeholders, including vulnerable populations. The plan describes the vulnerable populations within the jurisdiction and processes for providing services to the identified populations. | The RAND corporation independently reviewed the pilot sites when PPHR was first developed. Since  2004, more than 450 jurisdictions across the United States  have been covered by an agency recognized as meeting the PPHR requirements.[13,14] | Independent evaluation, consultation, annual program updates have helped to establish a valid and reliable program that is relevant and useful to LPHDs. | High |

| **Author, Year Title Purpose/Aim Country; Hazard Method of Development Indicator Themes PHEP Elements Ethics/Values Results Validation Quality Appraisal**  **Jurisdictional Level** | | | | | | | | | | | |
| --- | --- | --- | --- | --- | --- | --- | --- | --- | --- | --- | --- |
| Population Health and Wellness (British Columbia Ministry of  Health), 2005[15] | CORE Public Health Functions for  BC. Model Core Program Paper | The model core program paper is a resource for health authorities developing their core programs through a performance improvement planning process. | Canada; Local/Regional | All hazard | The evidence base from the literature was reviewed. A working group of representatives from the Ministry of Health and the health authorities worked together in the development of the paper. | Surveillance of health risks and vulnerabilities; Hazard, risk and vulnerability analysis; mitigation measures;  preparedness, emergency response and business  continuity planning; emergency response plan; communication and education measures; resource management; training and exercising; recovery;  health emergency preparedness; indicators for overall health emergency management; external capacity and support requirements; key success factors/system strategies; intersectoral collaboration and coordination assessment and evaluation of the Health Emergency Management Program. | All elements | Ethics and values were briefly mentioned with regards to gathering information on the vulnerabilities of the population and the availability of resources to respond to an emergency, including working with community networks to identify vulnerable groups of people and their locations. | The benchmarks and indicators were not evaluated, only developed. It was envisioned that the performance improvement process will be implemented over a course of several years. The Model Core Program is a part of the BC Core Functions in Public Health. This paper identifies the core elements that are provided by BC health authorities in the field of health emergency management. The elements may be modified to meet local context and needs. | No further validation outside of consultation with an expert  Working group. | Low |
| Public Health Agency of Canada, 2005[16] | Canada’s International Health Regulations Implementation Project: International Health Regulations (2005) Surveillance and Response Capacity Assessment | This document is an inventory that was developed for provinces/territories to use  to report to the Public Health Agency of Canada (PHAC). PHAC is the focal point for reporting to the World Health Organization regarding the International Health Regulations (IHR). | Canada; Federal | All hazard | Indicators were developed based on the reporting guidelines for the IHR. | General or overarching questions (e.g., legislation, plans, protocols, report capacity, etc.);  Report on indicators pertaining to five types of events: communicable disease, biological, chemical events, radio-nuclear events and zoonotic;  Each theme is subdivided into criteria of detection, reporting and notification, confirmation/verification, risk assessment and response;  Narrative questions (e.g., Given your current capacities (and work in progress) do you expect that your province/territory would be able to 1) detect, 2) implement an appropriate response and 3) report to Public Health Agency of Canada, to events that would meet the definition of a "public health emergency of international concern" at this point in time? yes/no, why; accomplishments to share); IHR inventory documents. | All elements | Not discussed | N/A | N/A | Moderate |

| **Author, Year Title Purpose/Aim Country; Hazard Method of Development Indicator Themes PHEP Elements Ethics/Values Results Validation Quality Appraisal**  **Jurisdictional Level** | | | | | | | | | | | |
| --- | --- | --- | --- | --- | --- | --- | --- | --- | --- | --- | --- |
| Center for Public Health Systems and Services Research, 2016[17] | National Health Security Preparedness Index Measures List. | The Index is designed to summarize levels of preparedness achieved within individual states and for the nation as a whole, with the goal of disseminating and using this information for policy directives and processes improvements. | USA; State | All hazard | The index was developed through consultation with 33 organizations, experts, institutions and health units using a Delphi method. Measurement (construct) validity and reliability analyses of the Index domains and subdomains was undertaken, including internal consistency reliability performed at the subdomain, domain, and overall Index level. Multi-trait scale analyses tests were performed at the subdomain and domain levels.  Sensitivity analyses were used to examine the relative influence of each measure on overall Index results, including the impact of the Index’s methods for scaling, imputing, and aggregating individual measures into subdomains, domains, and overall Index values. Ideas for new and modified measures were solicited through an Open Call for Measures and through monthly Index Workgroup meetings with content experts and stakeholders in the preparedness field. Index measures are updated every three years. | The Index consists of six domains: 1. Health security surveillance; 2. Community planning and engagement; 3. Information and incident management: actions to deploy people, supplies, money and information; 4. Healthcare delivery; 5. Countermeasure management: actions to store and deploy medical and pharmaceutical products that prevent and treat the effects of hazardous substances and infectious diseases; and, 6. Environmental and occupational health: actions to maintain the security and safety of water and food supplies, to test for hazards and contaminants in the environment, and to protect workers and emergency responders from health hazards. The Index further divides these six domains into 19 subdomains reflecting specific areas of practice and policy and an overall Index composite measure. All summary measures are scaled along a range from 0 to 10, with 10 representing the highest level of preparedness. The Index produces summary measures for each of the 51 states and for the whole nation. | Surveillance and monitoring; Community engagement; Workforce capacity; Collaborative networks; Communication; Resources. | Measures were developed according to the Pandemic and All- Hazards Preparedness Act (children, seniors, and pregnant women). Measures also include those requiring response assistance before, during, after an event (people with disabilities, live in institutionalized settings, from diverse cultures, have limited English proficiency, are transportation disadvantaged, have chronic medical disorders, and have pharmacological dependency). | The 2017 release of the Index is composed of scores for 139 individual measures, which includes 19  Foundational Capability measures. The previous version in 2016 contained 197 measures. A total of 58 measures were eliminated from the Index based on low construct validity, public comments and expert judgment. | Content validity; construct validity, and internal reliability of each domain and overall index. | High |
| Toner, 2017[18,19] | (1) A community Checklist for Health Sector Resilience Informed by Hurricane Sandy;  (2) Health Sector Resilience Checklist for High-Consequence Infectious Diseases—Informed by the Domestic US Ebola Response | To develop an evidence-informed checklist outlining action steps  for the health sector and public health authorities, in partnership with non-government organizations and private industry to strengthen community resilience to disasters and for emerging threats, such as Ebola virus and other high- consequence infectious diseases (HCID).  The health sector resilience checklist for HCID is a companion project of the community checklist for health sector resilience to disasters. | USA; Local/regional and State | CBRNE | A literature review was undertaken of all relevant peer- reviewed and grey literature sources related to the study; semi-structured interviews with 73 key informants from four cities that treated confirmed cases of EVD. Findings from the interview were discussed with an expert advisory group; semi-structured interviews with 67 key informants from the states impacted by Hurricane Sandy (New York, New Jersey, Connecticut and Pennsylvania). Interviews were followed by two focus group discussion to further explore themes emerging from the 67 interviews. | General checklist items: preparedness; leadership; creative flexibility; command structure;  health care coalitions; continuity of operations/  business continuity plan; communication, public awareness and situational awareness; engaging and supporting workers; public health legal preparedness; infrastructure, continuity and restoration; supply chains.  Public trust checklist items: managing uncertainty; crisis & emergency risk communication.  Public health checklist: public health issues; public health law; quarantine; monitoring programs; decontamination; waste management; healthcare and public health workforce issues; psychosocial effects; staffing levels & availability; voluntary vs mandatory staffing; clinical training, education, & drills; clinical personal protective equipment; laboratories; quality assurance & proficiency; laboratory personal protective equipment & training. | All elements | Whether staffing during an event should be voluntary or mandatory was a point of discussion - the recommendation of establishing an ethics committee to review such protocols was a recommendation.  The importance of public trust was identified and included in the findings. | The results of key informant interviews informed the development of the checklist. No further evaluation of the checklist was discussed. Five checklists were created as recommendations for public health, healthcare, EMS, and elected officials. An additional general checklist was developed for all sectors. These checklists form the Health Sector Resilience Checklist for Highly Infectious Diseases. | Engaging with stakeholders and an expert  advisory committee helped to establish content validity. No further validation was described. | Moderate |

| **Author, Year Title Purpose/Aim Country; Hazard Method of Development Indicator Themes PHEP Elements Ethics/Values Results Validation Quality Appraisal**  **Jurisdictional Level** | | | | | | | | | | | |
| --- | --- | --- | --- | --- | --- | --- | --- | --- | --- | --- | --- |
| WHO, 2013[20] | IHR Core Capacity Monitoring Framework: Checklist and Indicators for Monitoring Progress in the Development of IHR Core Capacities in State Parties. | This document proposes a framework and processes for state parties to monitor the development of their core capacities at the national, intermediate and community/primary response levels. The monitoring framework provides: 20 global indicators for monitoring the development of IHR core capacities for reporting annually to the WHA by all States Parties and other indicators for monitoring the comprehensive development, strengthening, and maintenance of core capacities. | International (196 countries and all WHO member states); Local/Regional, State and Federal | All hazard | The indicators were developed based on consensus of technical expert views drawn globally from WHO Member States, technical institutions, partners, and from within WHO in accordance with Annex 1  of the Regulations. The framework is based both on existing knowledge and on concepts and models that have been successfully applied in monitoring capacity development activities. It was developed according to three models: the Capability Maturation Index (CMI) model suggesting progressive levels of achievement; the Ripple Model which describes staged capacity building and the Potter’s model advocating the strengthening of existing structures, systems and institutional capacities. Details regarding development are not explained in this report. | Core capacity 1: National legislation, policy and financing; Core capacity 2: Coordination and national focal point communications; Core capacity 3: Surveillance; Core capacity 4: Response; Core capacity 5: Preparedness; Core capacity 6: Risk communication; Core capacity 7: Human resources; Core capacity 8: Laboratory. | All elements | Not discussed | This document does not report on the development or evaluation of the Checklist. Independent reviews and evaluations of IHR and various aspects of the Checklist and Indicators for Monitoring IHR progress have been published elsewhere.[21,22] | Not discussed | Moderate |
| WHO, 2012[23] | Strengthening Health System Emergency Preparedness: Toolkit For Assessing Health- System Capacity For Crisis Management. | The toolkit assists countries with assessing the capacity of their health systems to respond to various threats and identify gaps. The toolkit enables a ministry of health to record and classify information regarding its capacity to manage crises; establish responsibility for specific tasks; determine the relationship between those involved in these tasks (partners, sectors, disciplines) with the aim of synergizing resources; identify shortcomings and gaps; and monitor progress. | International (WHO Europe); Federal | All hazard | The toolkit is a product of the project called, “Support to health security, preparedness planning and crises management in European Union (EU), EU accession and neighboring (ENP) countries” developed by the European Commission Directorate-General for Health and Consumers and the WHO European Regional Office. It is based on the WHO health system framework, the Hospital safety index, Health-sector self-assessment tool for disaster risk reduction, and Protocol for assessing national surveillance and response capacities for the International Health Regulations (2005). The toolkit has been pilot tested in eight countries. It was developed through expert consultation to create a consensus set of guidelines and checklist. The toolkit consists of a “User manual” and the “Assessment form” and is subcategorized into 16 key components and 51 essential attributes. | Leadership and governance; health workforce; medical products, vaccines and technologies; health information; health financing; service delivery. | Governance and leadership; Collaborative networks; Practice and experience;  Workforce capacity;  Resources; Risk analysis; Surveillance and monitoring; Communication | Ethics and values were discussed in terms of having mechanisms in place to address cultural barriers and involving displaced populations in programmes. Such mechanisms should exist to ensure that health-care programmes are culturally appropriate, accessible and affordable. | [An evaluation of the toolkit has been undertaken independently by other researchers, including a study that drew from a qualitative analysis of the evidence and experience found in the literature. The findings support the tbuild resilience across the health system levers for an all- hazards approach to disaster management](http://currents.plos.org/disasters/article/developing-a-health-system-approach-to-disaster-management-a-qualitative-analysis-of-the-core-literature-to-complement-the-who-toolkit-for-assessing-health-system-capacity-for-crisis-management/). [24] | Not discussed | Moderate |

| **Author, Year Title Purpose/Aim Country; Hazard Method of Development Indicator Themes PHEP Elements Ethics/Values Results Validation Quality Appraisal**  **Jurisdictional Level** | | | | | | | | | | | |
| --- | --- | --- | --- | --- | --- | --- | --- | --- | --- | --- | --- |
| WHO, 2012[25] | Health indicators of disaster risk management in the context of the Rio+20 Conference on Sustainable Development. | This document proposes key indicators for disaster risk management. | International | All hazard | Brazil launched a National Commission to support the organization of the UN Sustainable Development Conference (Rio+20) consisting of a WHO Expert Consultation from 17–18 May 2012 in Geneva. The consultation included over 40 participants with expertise in health, equity, development and environment from research institutions, government, and multi-lateral/bilateral development agencies. The briefings also draw upon previous work by the WHO and WHO regional offices as well as WHO frameworks. The expert consultation was co-sponsored and supported by the National Institute of Environmental Health Sciences, USA. | Hazard impacts on human health and wellbeing; Reporting of disaster data on health impacts at a national level;  Assessment of emergency and disaster-related  Risks; Development planning to reduce health impacts of disasters; Safer, prepared and resilient health facilities; National health emergency risk management programmes; Health services for disasters (health coverage indicator);  International health regulations;  Community resilience;  Risk assessments and disaster-related data on  deaths, disease, and disabilities. | Governance and leadership Planning process Risk analysis Surveillance and monitoring Community engagement Resources | Equity was considered when accounting for variance in vulnerabilities, resilience, and response capacities related to gender, socio-economic factors, age, disability, mobility, social isolation, and ethnicity. | Disaster risk management forms part of the thematic areas of the Health Impact Assessment. Other areas include agriculture, food and nutrition security, energy, jobs, sustainable cities, and water. | Content validity was determined by expert consultation and drawing from previous work of the WHO. | Low |
| WHO, 2005[26] | Joint External Evaluation Tool: International health regulations (2005) | The purpose of the external evaluation process is to measure country specific status and progress in achieving the targets set out by the International Health Regulations (IHR). IHR is a legally binding international law that encourages countries to work together to prevent the spread of diseases and other health risks. | International; Federal | All hazard | Indicators were developed by technical  experts in accordance with the IHR. It was  based on existing knowledge and of concepts and models that have been successfully applied in monitoring capacity and development activities. No further details about the development processes were discussed. | National legislation, policy and financing;  IHR coordination, communication and advocacy; antimicrobial resistance;  zoonotic disease; food safety;  biosafety and biosecurity; immunization;  national laboratory system; real time surveillance; reporting; workforce development;  preparedness (planning) risk mapping; emergency response operations; linking public health and security authorities; medical countermeasures and personnel deployment; risk Communication;  points of entry; chemical events; radiation emergencies. | All elements | Not discussed. | Countries use self- reported data to respond to the indicators. The Joint External Evaluation Tool team reviews the data and then conducts a site visit for in-depth discussion. Afterwards the team prepares a report to share with the country, and with permission, other stakeholders to guide  best practices, lessons learned, and future IHR developments. | Not discussed | Low |

**References**

1. Asch SM, Stoto M, Mendes M, Valdez RB, Gallagher ME, Halverson P, et al. A review of instruments assessing public health preparedness. Public Health Rep. 2005;120(5):532-542.

2. Babaie J, Ardalan A, Vatandoost H, Goya MM, Akbarisari A. Developing a performance assessment framework and indicators for communicable disease management in natural disasters. Prehosp Disaster Med. 2016;31(1):27-35.

3. Barnett DJ, Balicer RD, Lucey DR, Everly GS, Omer SB, Steinhoff MC, et al. A systematic analytic approach to pandemic influenza preparedness planning. PLoS Med. 2005;2(12):e359.

4. Dorn BC, Savoia E, Testa MA, Stoto MA, Marcus LJ. Development of a survey instrument to measure connectivity to evaluate national public health preparedness and response performance. Public Health Rep. 2007;122(3):329-338.

5. Gebbie KM, Valas J, Merrill J, Morse S. Role of exercises and drills in the evaluation of public health in emergency response. Prehosp Disaster Med. 2006;21(3):173-182. doi:10.1017/S1049023X00003642.

6. Harvard TH Chan School of Public Health. Emergency preparedness exercise evaluation toolkit. Harvard TH Chan School of Public Health Web site. Available: [https://phasevtechnologies.com/studies/
lamps/index.php](https://phasevtechnologies.com/studies/lamps/index.php).

7. Agboola F, Bernard D, Savoia E, Biddinger PD. Development of an online toolkit for measuring performance in health emergency response exercises. Prehosp Disaster Med. 2015;30(5):503-508.

8. Lee AV, Vargo J, Seville E. Developing a tool to measure and compare organizations’ resilience. Nat Hazards Rev. 2013;14(1):29-41.

9. Lis R, Sakata V, Lien O. How to choose? Using the Delphi method to develop consensus triggers and indicators for disaster response. Disaster Med Public Health Prep. 2017;11(4):467-472.

10. Mann NC, MacKenzie E, Anderson C. Public health preparedness for mass-casualty events: a 2002 state-by-state assessment. Prehosp Disaster Med. 2004;19(3):245-255.

11. Centers for Disease Control and Prevention (CDC); U.S. Department of Health and Human Services. Public Health Preparedness Capabilities: National Standards for State and Local Planning. Atlanta, GA: Centers for Disease Control and Prevention; 2011. Available: [https://www.cdc.gov/cpr/readiness/
00_docs/DSLR_capabilities_July.pdf](https://www.cdc.gov/cpr/readiness/00_docs/DSLR_capabilities_July.pdf).

12. National Association of County and City Health Officials (NACCHO). Project Public Health Ready criteria: version 8.1. Available: <https://www.naccho.org/uploads/downloadable-resources/PPHR-Criteria-Version-8.1.pdf>.

13. Myers S, Stoto M. Process Evaluation of Project Public Health Ready. Santa Monica, CA: RAND Corporation; 2005. Available: <https://www.rand.org/pubs/technical_reports/TR224.html>.

14. Estrada LC, Fraser MR, Cioffi JP, Sesker D, Walkner L, Brand MW, et al. Partnering for preparedness: the Project Public Health Ready experience. Public Health Rep. 2005;120(suppl 1):69-75.

15. Population Health and Wellness; Ministry of Health Services; Province of British Columbia. A Framework for Core Functions in Public Health: Resource Document. Victoria, BC: Government of British Columbia; 2005. Available: <https://www.health.gov.bc.ca/library/publications/year/2005/core_functions.pdf>.

16. Public Health Agency of Canada. Canada's international health regulations implementation project: international health regulations (2005): surveillance and response capacity assessment [Excel file]. Accessed upon request December 6, 2018.

17. Center for Public Health Systems and Services Research. National Health Security Preparedness Index Measures List: April 2016 Release. Lexington, KY: University of Kentucky; 2016. Available: <https://nhspi.org/wp-content/uploads/2016/12/NHSPI-Measures-List_April-2016-Release.pdf>.

18. Toner ES, McGinty M, Schoch-Spana M, Rose DA, Watson M, Echols E, et al. A community checklist for health sector resilience informed by Hurricane Sandy. Health Secur. 2017;15(1):53-69.

19. Toner E, Shearer M, Sell T, Meyer D, Chandler H, Schoch-Spana M, et al; Centers for Disease Control and Prevention (CDC); Johns Hopkins Center for Health Security. Health Sector Resilience Checklist for High-Consequence Infectious Diseases--Informed by the Domestic US Ebola Response. Baltimore, MD: John Hopkins Center for Health Security; 2017. Available: <http://www.centerforhealthsecurity.org/our-work/pubs_archive/pubs-pdfs/2017/HCID_Final_Report_05.23.2017.pdf>.

20. World Health Organization. IHR Core Capacity Monitoring Framework: Checklist and Indicators for Monitoring Progress in the Development of IHR Core Capacities in State Parties. Geneva, Switzerland: World Health Organization; 2013. Available: <http://www.who.int/ihr/checklist/en/>.

21. World Health Organization. IHR news: the WHO quarterly bulletin on IHR implementation: what's new in IHR coordination and support. December 29, 2011. Available: [https://www.who.int/ihr/
ihrnews/IHR_news_No_17.pdf?ua=1](https://www.who.int/ihr/ihrnews/IHR_news_No_17.pdf?ua=1).

22. Gostin LO, Katz R. The international health regulations: the governing framework for global health security. Milbank Q. 2016;94(2):264-313.

23. World Health Organization Regional Office for Europe. Strengthening Health System Emergency Preparedness: Toolkit for Assessing Health System Capacity for Crisis Management. Copenhagen, Denmark: World Health Organization; 2012. Available: [http://www.euro.who.int/__data/assets/pdf_file
/0008/157886/e96187.pdf](http://www.euro.who.int/__data/assets/pdf_file/0008/157886/e96187.pdf).

24. Bayntun C, Rockenschaub G, Murray V. Developing a health system approach to disaster management: a qualitative analysis of the core literature to complement the WHO toolkit for assessing health-system capacity for crisis management. PLoS Curr.2012;4:e5028b6037259a.

25. World Health Organization. Health indicators of disaster risk management in the context of the Rio+20 UN conference on sustainable development: initial findings from a WHO expert consultation: 17‐18 May 2012. Available: <http://www.who.int/hia/green_economy/indicators_disasters1.pdf>.

26. World Health Organization. Joint External Evaluation Tool: International Health Regulations (2005). Geneva, Switzerland: World Health Organization; 2016. Available: <http://apps.who.int/iris/bitstream/handle/10665/204368/9789241510172_eng.pdf;jsessionid=C209D8B77D2C092A2CECD2AFD5DDAB08?sequence=1>.
