# Supplementary material for: Are we prepared? The development of performance indicators for public health emergency preparedness using a modified Delphi approach
Source: PLoS One. 2019 Dec 23;14(12):e0226489. doi: 10.1371/journal.pone.0226489 (PMC6927653; doi:10.1371/journal.pone.0226489)
Supplement: S2 Appendix — (DOCX) [file pone.0226489.s004.docx]

# S4 Appendix: Indicators

# S4 Table 1. Round one survey indicators that achieved ≥ 70% consensus level on both criteria

|  | **Important** | | | **Actionable** | | |
| --- | --- | --- | --- | --- | --- | --- |
| **Indicators (n=41)** | **Median (IQR)** | **No. (n=33)** | **Consensus**  **Level (%)** | **Median (IQR)** | **No. (n=33)** | **Consensus**  **Level (%)** |
| **Governance and Leadership** |  |  |  |  |  |  |
| 1. The public health agency aligns its emergency plans and/or protocols with provincial, territorial and/or federal policy on public health and emergency management. | 6 (1) | 31 | 93.9 | 6 (1) | 31 | 93.9 |
| 2. The public health agency is a member of a local/regional multidisciplinary structure that aims to reduce community risks to emergencies and disasters. Network partners involved in this structure may include transportation, planners, industry, local/regional elected officials. | 6 (1) | 31 | 93.9 | 5 (2) | 24 | 72.7 |
| 3. The public health agency is a member of a local/regional structure for health-sector emergency management that aims to coordinate health system preparedness for emergencies. Network partners involved in this structure may include; for example, acute care, primary care, or emergency medical services, depending on the jurisdiction. | 7 (1) | 32 | 97.0 | 6 (2) | 29 | 87.9 |
| 4. The public health agency’s policies describe the authority and procedures under which it would respond to an emergency in a supportive role. | 6 (1) | 32 | 97.0 | 6 (2) | 28 | 84.8 |
| 5. The public health agency’s policies describe the authority and procedures under which it would respond to an emergency in a supportive role to the lead agency. | 6 (1) | 31 | 93.9 | 6 (0) | 29 | 87.9 |
| 6. The public health agency’s policies define the conditions and procedures for using incident management structures and processes to coordinate agency activities in emergencies. | 6 (1) | 32 | 97.0 | 6 (2) | 27 | 81.8 |
| 8. The public health agency’s policies define processes for establishing a clear leader in the context of emergency. | 7 (1) | 30 | 90.9 | 6 (2) | 26 | 78.8 |
| **Planning Process** |  |  |  |  |  |  |
| 9. The roles and responsibilities of the public health agency for responding to all-hazards emergencies are defined in agency plans and/or protocols. | 7 (1) | 31 | 93.9 | 6 (1) | 30 | 90.9 |
| 10. The public health agency’s emergency management plans and/or protocols relate to all phases of a disaster (E.g. Prevention/mitigation, preparedness, response, and recovery). | 6 (1) | 28 | 84.8 | 6 (2) | 28 | 84.8 |
| 11. The roles and responsibilities for the public health agency in ensuring business continuity during an emergency are established in agency plans and/or protocols. | 6 (1) | 31 | 93.9 | 6 (1) | 31 | 93.9 |
| 12. Linkages between the public health agency and network partners’ emergency plans and/or protocols are discussed with involved network partners. | 6 (1) | 28 | 84.8 | 6 (2) | 24 | 72.7 |
| 13. The public health agency has a process to support priority-setting decisions in the allocation of limited resources in the context of emergencies. | 6 (1) | 29 | 87.9 | 6 (3) | 24 | 72.7 |
| **Risk Analysis** |  |  |  |  |  |  |
| 14. The public health agency conducts a comprehensive risk assessment for all-hazards emergencies at regular intervals (E.g. Annually, or when a new threat is identified), to adapt to emerging risks. | 6 (2) | 28 | 84.8 | 6 (1) | 27 | 81.8 |
| 15. The public health agency uses locally relevant data to inform risk assessment. Examples of data sources may include: communicable diseases, vector-borne diseases, food and water testing, population health determinants, non-communicable diseases such as injuries. | 6 (1) | 31 | 93.9 | 6 (1) | 27 | 81.8 |
| 16. The public health agency’s risk assessment process includes an analysis of organizational capacity to manage the identified risks. | 6 (1) | 32 | 97.0 | 6 (1) | 29 | 87.9 |
| 17. The public health agency’s risk assessment process considers the preparedness capacity of populations that may be at increased risk in the context of emergencies. | 6 (2) | 28 | 84.8 | 6 (2) | 24 | 72.7 |
| 18. The public health agency uses the results of the risk assessment to inform relevant plans/protocols for emergency management, business continuity and/or risk reduction. | 6 (1) | 32 | 97.0 | 6 (1) | 30 | 90.9 |
| **Resources** |  |  |  |  |  |  |
| 20. The public health agency has dedicated financial resources to support planning and preparedness activities for emergencies. | 7 (2) | 28 | 84.8 | 5 (2) | 24 | 72.7 |
| 22. The public health agency has established procedures to facilitate timely dispensing of physical resources to the community in the context of emergencies (E.g. This may include medical prophylaxis and/or treatment). | 6 (1) | 32 | 97.0 | 6 (2) | 29 | 87.9 |
| **Collaborative Networks** |  |  |  |  |  |  |
| 24. The public health agency has mechanisms for contacting network partners in the event of an emergency. | 7 (1) | 33 | 100 | 6 (1) | 30 | 90.9 |
| 27. The public health agency has demonstrated the ability to perform cooperative activities with network partners. This ability may be demonstrated, for instance, during real or simulated emergencies. | 6 (1) | 32 | 97.0 | 6 (1) | 27 | 81.8 |
| **Community Engagement (none)** |  |  |  |  |  |  |
| **Communication** |  |  |  |  |  |  |
| 33. The public health agency has developed communication strategies for multiple audiences in advance of emergencies, based on its risk assessment. | 6 (2) | 30 | 90.9 | 6 (1) | 29 | 87.9 |
| 34. The public health agency communication strategy uses multiple communication platforms to facilitate timely information-sharing in the context of an emergency; for example, town-hall meetings, websites, social media, spokespersons, information call lines/centres. | 7 (1) | 31 | 93.9 | 6 (1) | 32 | 97.0 |
| 35. The public health agency has a process for the public and media to ask questions and voice concerns; for example, town hall meetings, social media, information call lines/centres. | 7 (1) | 30 | 90.9 | 6 (1) | 29 | 87.9 |
| 36. The public health agency has a process for monitoring the media, including social media, to rapidly identify rumours and correct misinformation. | 6 (1) | 31 | 93.9 | 6 (1) | 26 | 78.8 |
| 37. The public health agency has structures to ensure message consistency with network partners; for example, regular network partner coordination meetings, incident management systems. | 7 (1) | 33 | 100 | 5 (2) | 24 | 72.7 |
| 38. The public health agency has capacity for redundancy in communication platforms in the context of an emergency; for example, using alternate platforms in power outages or if regular communication channels are down. | 6 (1) | 32 | 97.0 | 5 (1) | 26 | 78.8 |
| **Workforce Capacity** |  |  |  |  |  |  |
| 41. The public health agency has a workforce professional development plan for training its staff that is specific to emergency management topics; for example, content of emergency plans/protocols, incident management systems, communications. | 6 (1) | 30 | 90.9 | 6 (1) | 25 | 75.8 |
| 42. The public health agency conducts needs assessments regularly to determine the emergency management training needs of its workers. | 6 (2) | 26 | 78.8 | 5 (2) | 24 | 72.7 |
| 43. The public health agency has an up-to-date inventory of staff trained in emergency management topics; for example, content of emergency plans/protocols, incident management systems, communications. | 6 (2) | 27 | 81.8 | 6 (1) | 28 | 84.8 |
| 45. The public health agency workforce has demonstrated the ability to perform cooperative activities as an organization in the context of emergencies. This may be demonstrated, for instance, during exercises or activations. | 6 (1) | 30 | 90.9 | 5 (1) | 25 | 75.8 |
| 46. The public health agency has established policies and procedures for supporting staff during an emergency with respect to their health and wellbeing; for example, on personal safety, mental well-being, family commitments. | 7 (1) | 30 | 90.9 | 6 (2) | 29 | 87.9 |
| **Surveillance and Monitoring** |  |  |  |  |  |  |
| 49. The public health agency uses a syndromic surveillance and/or other early warning systems to detect potential public health emergencies in a timely manner. | 6 (1) | 30 | 90.9 | 5 (3) | 24 | 72.7 |
| 51. The public health agency has the capability to conduct rapid health risks and/or needs assessments for communities recently impacted by emergencies. | 6 (2) | 29 | 87.9 | 5 (2) | 24 | 72.7 |
| **Learning and Evaluation** |  |  |  |  |  |  |
| 53. The public health agency applies a self-assessment process to emergency management. This process may be applied to tests, exercises, simulations and/or emergency plan activations and agency responses. | 6 (1) | 28 | 84.8 | 6 (2) | 28 | 84.8 |
| 54. The public health agency’s self-assessment process is used to identify capabilities, strengths and/or assets to describe successes relevant to emergency management. | 6 (2) | 28 | 84.8 | 6 (2) | 27 | 81.8 |
| 55. The public health agency’s self-assessment process is used to inform improvement actions; for example, identifying responsible groups for corrective actions and establishing timelines for change. | 6 (1) | 28 | 84.8 | 6 (2) | 26 | 78.8 |

|  | **Important** | | | **Actionable** | | |
| --- | --- | --- | --- | --- | --- | --- |
| **Indicators (n=41)** | **Median (IQR)** | **No. (n=33)** | **Consensus**  **Level (%)** | **Median (IQR)** | **No.**  **(n=33)** | **Consensus**  **Level (%)** |
| **Practice and Experience** |  |  |  |  |  |  |
| 58. The public health agency conducts regular needs assessments to determine the needs for organizational practice of emergency plans and/or protocols; for example, the emergency response plan, the business continuity plan. The assessment may consider recent table tops, exercises, simulations, or activations in response to emergencies. | 6 (2) | 31 | 93.9 | 6 (1) | 27 | 81.8 |
| 60. The public health agency practices its plans and/or protocols that are relevant to emergency management; for example, the agency emergency response plan, the business continuity plan. Practice may include table tops, exercises, simulations, or activations for emergencies. | 7 (1) | 31 | 93.9 | 6 (2) | 28 | 84.8 |
| 61. Public health agency practice of emergency management activities (e.g., table tops, exercises, simulations) includes the regular attendance of both management and staff. | 6 (2) | 28 | 84.8 | 6 (2) | 27 | 81.8 |
| 62. Public health agency management and staff have demonstrated the ability to adjust plans and/or protocols for emergencies in the context of new knowledge, uncertain science, and/or differences in professional opinions. This ability may be demonstrated during real or simulated emergencies. | 6 (2) | 31 | 93.9 | 6 (1) | 25 | 75.8 |

# S4 Table 2. Round one survey indicators with unclear consensus (≤ 70%) on one or both criteria

|  | **Important** | | | **Actionable** | | |
| --- | --- | --- | --- | --- | --- | --- |
| **Indicators (n=21)** | **Median (IQR)** | **No. (n=33)** | **Consensus**  **Level (%)** | **Median (IQR)** | **No. (n=33)** | **Consensus**  **Level (%)** |
| **Governance and Leadership** |  |  |  |  |  |  |
| 7. The public health agency’s policies define requirements for reporting to the provincial/territorial and/or federal public health authority on community health risks in the context of an emergency; for example, radio-nuclear, chemical or biosecurity events. | 6 (1) | 29 | 87.9 | 6 (3) | 19 | 57.6 |
| **Resources** |  |  |  |  |  |  |
| 19. The public health agency has a dedicated emergency preparedness coordinator, or similar position, led by an individual that has completed a training program in emergency management. | 7 (1) | 29 | 87.9 | 6 (3) | 23 | 69.7 |
| 21. The public health agency has a system in place to support management of physical resources relevant to emergencies; for example, equipment, supplies or medical prophylaxis and/or treatment (E.g. This may include tracking, monitoring and/or reporting components). | 6 (2) | 29 | 87.9 | 6 (2) | 23 | 69.7 |
| 23. The public health agency has established procedures for the exceptional procurement of physical resources relevant to the emergency context; for example, equipment, supplies or medical prophylaxis and/or treatment from the provincial, territorial or federal government. | 6 (1) | 29 | 87.9 | 6 (3) | 20 | 60.6 |
| **Collaborative Networks** |  |  |  |  |  |  |
| 25. The public health agency has mutual aid agreements in place with network partners that describe how resources and/or services will be shared during an emergency, including meeting demands for surge capacity. | 6 (1) | 27 | 81.8 | 5 (2) | 20 | 60.6 |
| 26. The public health agency has partnerships to access specialized expertise relevant to community risks; for example, environmental health, biosecurity, toxicology, transportation companies, legal advice. | 6 (1) | 30 | 90.9 | 5 (3) | 22 | 66.7 |
| 28. The public health agency has established processes to effectively manage differences and disputes with network partners in the context of an emergency. | 6 (2) | 26 | 78.8 | 5 (2) | 18 | 54.5 |
| **Community Engagement** |  |  |  |  |  |  |
| 29. The public health agency provides education programs directed at the public to raise awareness about preparedness for relevant community risks. | 6 (2) | 26 | 78.8 | 5 (2) | 21 | 63.6 |
| 30. The public health agency has an established structure to facilitate inclusion of community considerations in public health emergency management; for example, a community advisory committee that includes members of the public and/or advocacy groups that represent the public. | 5 (3) | 20 | 60.6 | 5 (2) | 23 | 69.7 |
| 31. The public health agency dedicates time for the continuous development of relationships with community organizations relevant to preparedness for local risks; for example, building relationships with members of the public and/or advocacy groups that represent the public. | 6 (2) | 24 | 72.7 | 6 (2) | 23 | 69.7 |
| 32. The public health agency has a process to ensure transparency with the public in cases where there are genuine differences of professional opinion in the context of uncertain science. | 6 (2) | 24 | 72.7 | 4 (1) | 16 | 48.5 |
| **Communication** |  |  |  |  |  |  |
| 39. The public health agency has access to dedicated communications personnel for emergencies. | 7 (1) | 31 | 93.9 | 6 (4) | 22 | 66.7 |
| **Workforce Capacity** |  |  |  |  |  |  |
| 40. The public health agency has a structure that supports multi-disciplinary emergency management across the agency; for example, a multi-disciplinary team of public health professionals, epidemiologists, environmental health officers. | 6 (1) | 29 | 87.9 | 6 (3) | 23 | 69.7 |
| 44. The public health agency workforce is available for the management of emergencies on a 24/7/365 basis. | 6 (1) | 29 | 87.9 | 5 (4) | 23 | 69.7 |
| 47. The public health agency has a process for integrating volunteers into public health service delivery in emergency situations. | 5 (2) | 24 | 72.7 | 5 (2) | 21 | 63.6 |
| **Surveillance and Monitoring** |  |  |  |  |  |  |
| 48. The public health agency has the capability for enhanced and/or event-based surveillance specific to local/regional risks. | 7 (1) | 29 | 87.9 | 5 (2) | 23 | 69.7 |
| 50. The public health agency surveillance system has the capacity for data-sharing with other relevant surveillance systems; for example, with agricultural, veterinary or environmental surveillance systems. | 6 (1) | 26 | 78.8 | 4 (2) | 15 | 45.5 |
| 52. Local/regional laboratories (e.g., commercial and/or hospital) have the capability for timely reporting of results to the public health agency for surveillance relevant to public health emergencies. | 6 (1) | 32 | 97.0 | 5 (3) | 19 | 57.6 |
| **Learning and Evaluation** |  |  |  |  |  |  |
| 56. The public health agency has defined a research and/or evaluation agenda that aims to address knowledge gaps relevant to emergency management. | 6 (2) | 24 | 72.7 | 5 (3) | 20 | 60.6 |
| 57. The public health agency has allocated resources for research and/or evaluation to address knowledge gaps relevant to emergency management. | 6 (3) | 22 | 66.7 | 5 (2) | 18 | 54.5 |
| **Practice and Experience** |  |  |  |  |  |  |
| 59. The public health agency has sufficient resources to practice plans and/or protocols relevant to emergency management; for example, the emergency response plan, the business continuity plan. Practice may include table tops, exercises, or simulations. | 6 (2) | 27 | 81.8 | 5 (3) | 19 | 57.6 |

# S4 Table 3. Indicators suggested by the panel in the round one survey

| PHEP framework element | Suggested indicator |
| --- | --- |
| Governance and leadership | The public health agency has defined leadership competencies for individuals that may act as agency leaders in an emergency. These may include: established effective relationships, local knowledge, credible, flexible, trusted, ethical. |
|  | The public health agency’s policies describe the roles and responsibilities of network partners in vertical or horizontal multi-jurisdictional response to emergencies; for example, responsibilities for different levels of government. |
|  | The public health agency’s policies define the conditions and procedures for escalating response to an emergency, including processes for declaring an event multi-jurisdictional. |
|  | The public health agency engages with policy-makers to address gaps in policy and/or legislation that pertain to the effectiveness of its emergency management plans and/or protocols. |
|  | Provincial/territorial policies and/or structure define roles and responsibilities for local/regional public health agencies in public health emergency management. |
| Planning process | The public health agency reviews its emergency plans and/or protocols with involved departments and/or programs internal to the agency. |
| Resources | The public health agency is able to secure financial resources to support response to and recovery from an emergency. |
| Collaborative  networks | The public health agency and its network partners have demonstrated shared understanding of roles, responsibilities and procedures relevant to the emergency context. This understanding may be demonstrated, for instance, during real or simulated emergencies. |
|  | The public health agency has mutual aid agreements in place with network partners outside the health sector that describe how resources and/or services will be shared during an emergency, including meeting demands for surge capacity; for example, non-governmental organizations such as the Red Cross. |
| Community engagement | The public health agency and/or its network partners engage with Indigenous communities regarding emergencies and related risks. Engagement may include community-specific risk assessments, plans and/or protocols, and inclusion of traditional knowledge where possible and appropriate. |
| Communication | The public health agency has a mechanism to formally or informally coordinate joint messaging with relevant network partners in a timely manner. |
|  | The public health agency communication strategy includes procedures for directly reaching citizens during an emergency, if required. For example, door-to-door, giving out pamphlets, engaging in informal street/neighbourhood gatherings. |
|  | The public health agency communication strategy includes plans and/or procedures for ensuring cultural competency and/or sensitivity to impacted communities for relevant risks and the emergency context. This includes procedures for translation of messages to relevant languages. |
|  | The public health agency has identified trained spokesperson(s) for the agency relevant to community risks and the emergency context. |

# S4 Table 4. Round two survey indicators that achieved ≥ 70% consensus level on both criteria

|  | **Important** | | | **Actionable** | | |
| --- | --- | --- | --- | --- | --- | --- |
| **Indicators (n=23)^a^** | **Median (IQR)** | **No. (n=33)** | **Consensus**  **Level (%)** | **Median (IQR)** | **No. (n=33)** | **Consensus Level (%)** |
| **Governance and Leadership** |  |  |  |  |  |  |
| 7R. The public health agency’s policies align with requirements for reporting to the provincial/territorial and/or federal public health authority on community health risks in the context of an emergency; for example, radio-nuclear, chemical or biosecurity events. | 6 (2) | 31 | 93.9 | 5 (1) | 28 | 84.8 |
| 1N. The public health agency has defined leadership competencies for individuals that may act as agency leaders in an emergency. These may include: established effective relationships, local knowledge, credible, flexible, trusted, ethical. | 6 (1) | 28 | 84.8 | 6 (1) | 25 | 75.8 |
| 3N. The public health agency’s policies define the conditions and procedures for escalating response to an emergency, including processes for declaring an event multi-jurisdictional. | 6 (1) | 31 | 93.9 | 6 (1) | 25 | 75.8 |
| **Planning Process** |  |  |  |  |  |  |
| 6N. The public health agency reviews its emergency plans and/or protocols with involved departments and/or programs internal to the agency. | 6 (1) | 33 | 100 | 6 (1) | 33 | 100 |
| **Resources** |  |  |  |  |  |  |
| 19R. The public health agency has or has access to a dedicated emergency preparedness coordinator, or similar position, led by an individual experienced in emergency management. | 7 (1) | 32 | 97.0 | 5 (1) | 26 | 78.8 |
| 21R. The public health agency has or has access to a system to support management of physical resources relevant to emergencies; for example, equipment, supplies or medical prophylaxis and/or treatment (e.g., this may include tracking, monitoring and/or reporting components). | 6 (1) | 31 | 93.9 | 5 (1) | 25 | 75.8 |
| 23R. The public health agency is familiar with established procedures for the exceptional procurement of physical resources relevant to the emergency context, including procedures for procurement outside of business hours; for example, equipment, supplies or medical prophylaxis and/or treatment from the provincial, territorial or federal government. | 6 (1) | 30 | 90.9 | 6 (1) | 25 | 75.8 |
| **Collaborative Networks** |  |  |  |  |  |  |
| 25R. The public health agency has mutual aid agreements in place with health-sector network partners that describe how resources and/or services will be shared during an emergency, including meeting demands for surge capacity. | 6 (2) | 31 | 93.9 | 6 (2) | 24 | 72.7 |
| 26R. The public health agency has partnerships and/or mechanisms to access to specialized expertise relevant to community risks; for example, environmental health, biosecurity, toxicology, transportation companies, legal advice. | 6 (1) | 32 | 97.0 | 6 (2) | 24 | 72.7 |
| **Community Engagement** |  |  |  |  |  |  |
| 29R. The public health agency provides and/or endorses education programs directed at the public to raise awareness about preparedness for relevant community risks. | 6 (1) | 30 | 90.9 | 5 (1) | 28 | 84.8 |
| 30R. The public health agency has or participates in an established structure to facilitate inclusion of community considerations in relevant aspects of public health emergency management. For example, a community advisory committee to inform emergency mitigation, planning and/or recovery including members of the public and/or advocacy groups that represent the public. | 5 (1) | 27 | 81.8 | 5 (1) | 27 | 81.8 |
| 31R. The public health agency dedicates time for the continuous development of relationships with community organizations relevant to preparedness for local risks and the agency context; for example, building relationships with members of the public and/or advocacy groups that represent the public. | 6 (1) | 27 | 81.8 | 6 (1) | 28 | 84.8 |
| 10N. The public health agency and/or its network partners engage with Indigenous communities regarding emergencies and related risks. Engagement may include community-specific risk assessments, plans and/or protocols, and inclusion of traditional knowledge where possible and appropriate. | 6 (1) | 27 | 81.8 | 5 (3) | 24 | 72.7 |
| **Communication** |  |  |  |  |  |  |
| 39R. The public health agency has access to communications personnel that are dedicated to the emergency and appropriately trained in crisis communication. | 6 (1) | 31 | 93.9 | 5 (1) | 28 | 84.8 |
| 11N. The public health agency has a mechanism to formally or informally coordinate joint messaging with relevant network partners in a timely manner. | 6 (1) | 33 | 100 | 6 (1) | 27 | 81.8 |
| 12N. The public health agency communication strategy includes procedures for directly reaching citizens during an emergency, if required. For example, door-to-door, giving out pamphlets, engaging in informal street/neighbourhood gatherings. | 6 (2) | 27 | 81.8 | 6 (1) | 26 | 78.8 |
| 13N. The public health agency communication strategy includes plans and/or procedures for ensuring cultural competency and/or sensitivity to impacted communities for relevant risks and the emergency context. This includes procedures for translation of messages to relevant languages. | 7 (1) | 30 | 90.9 | 6 (2) | 29 | 87.9 |
| 14N. The public health agency has identified trained spokesperson(s) for the agency relevant to community risks and the emergency context. | 6 (1) | 31 | 93.9 | 6 (1) | 30 | 90.9 |
| **Workforce Capacity** |  |  |  |  |  |  |
| 40R. The public health agency has a structure and/or mechanism to support multi-disciplinary emergency management relevant to community risks; for example, a multi-disciplinary team of public health professionals, epidemiologists, and environmental health officers. | 7 (1) | 30 | 90.9 | 6 (1) | 27 | 81.8 |
| 44R. The public health agency has a roster of its workforce available for the management of, or potential for, emergencies on a 24/7/365 basis. | 7 (1) | 32 | 97.0 | 6 (1) | 31 | 93.9 |
| **Surveillance and Monitoring** |  |  |  |  |  |  |
| 48R. The public health agency has the capability for or access to enhanced and/or event-based surveillance systems relevant to local/regional risks. | 7 (1) | 33 | 100 | 6 (3) | 24 | 72.7 |
| 50R. The public health agency has protocols and/or processes for information-sharing with network partners for purposes of surveillance of relevant risks; for example, with agricultural, veterinary or environmental surveillance systems. | 6 (1) | 31 | 93.9 | 5 (1) | 26 | 78.8 |
| **Practice and Experience** |  |  |  |  |  |  |
| 59R. The public health agency has sufficient resources to practice plans and/or protocols relevant to emergency management; for example, the emergency response plan, the business continuity plan. Practice may include table tops, exercises, or simulations. | 7 (1) | 29 | 87.9 | 6 (1) | 25 | 75.8 |

^a^R denotes a **revised** indicator; N denotes a **new** indicator suggested by the panel

# S4 Table 5. Round two survey indicators with unclear consensus (≤ 70%) on one or both criteria

|  | **Important** | | | **Actionable** | | |
| --- | --- | --- | --- | --- | --- | --- |
| **Indicators (n=12)^a^** | **Median (IQR)** | **No. (n=33)** | **Consensus**  **Level (%)** | **Median (IQR)** | **No. (n=33)** | **Consensus Level (%)** |
| **Governance and Leadership** |  |  |  |  |  |  |
| 2N. The public health agency’s policies describe the roles and responsibilities of network partners in vertical or horizontal multi-jurisdictional response to emergencies; for example, responsibilities for different levels of government. | 6 (1) | 29 | 87.9 | 5 (2) | 23 | 69.7 |
| 4N. The public health agency engages with policy-makers to address gaps in policy and/or legislation that pertain to the effectiveness of its emergency management plans and/or protocols. | 6 (2) | 28 | 84.8 | 5 (3) | 19 | 57.6 |
| 5N. Provincial/territorial policies and/or structure define roles and responsibilities for local/regional public health agencies in public health emergency management. | 6 (2) | 26 | 78.8 | 5 (3) | 17 | 51.5 |
| **Resources** |  |  |  |  |  |  |
| 7N. The public health agency is able to secure financial resources to support response to and recovery from an emergency. | 6 (1) | 29 | 87.9 | 4 (3) | 14 | 42.4 |
| **Collaborative Networks** |  |  |  |  |  |  |
| 28R. The public health agency has established processes, in cooperation with network partners, to effectively manage differences and disputes in the context of an emergency. | 5 (2) | 20 | 60.6 | 5 (1) | 18 | 54.5 |
| 8N. The public health agency and its network partners have demonstrated shared understanding of roles, responsibilities and procedures relevant to the emergency context. This understanding may be demonstrated, for instance, during real or simulated emergencies. | 6 (1) | 29 | 87.9 | 5 (3) | 22 | 66.7 |
| 9N. The public health agency has mutual aid agreements in place with network partners outside the health sector that describe how resources and/or services will be shared during an emergency, including meeting demands for surge capacity; for example, non-governmental organizations such as the Red Cross. | 6 (1) | 26 | 78.8 | 5 (3) | 20 | 60.6 |
| **Community Engagement** |  |  |  |  |  |  |
| 32R. The public health agency has a process to ensure transparency with the public with respect to what is known, what is not known and where there are genuine differences of professional opinion; for example, in the context of uncertain science or differences with Indigenous knowledge. | 6 (2) | 22 | 66.7 | 5 (2) | 21 | 63.6 |
| **Workforce Capacity** |  |  |  |  |  |  |
| 47R. The public health agency has a process and/or mechanism for integrating volunteers into emergency response and recovery. This may include volunteer recruitment, identification, credentialing and/or training processes and may be linked to processes in the broader health-sector. | 5 (2) | 19 | 57.6 | 5 (2) | 20 | 60.6 |
| **Surveillance and Monitoring** |  |  |  |  |  |  |
| 52R. Local/regional laboratories (e.g., commercial and/or hospital) have the capability for timely reporting of results to the public health agency for surveillance relevant to public health emergencies. | 7 (1) | 33 | 100 | 6 (3) | 21 | 63.6 |
| **Learning and Evaluation** |  |  |  |  |  |  |
| 56R. The public health agency has defined an evaluation, learning and/or research agenda that aims to address knowledge gaps relevant to emergency management. | 5 (2) | 21 | 63.6 | 5 (2) | 20 | 60.6 |
| 57R. The public health agency has resources allocated and/or identified for evaluation, learning and/or research to address knowledge gaps relevant to emergency management. | 6 (2) | 23 | 69.7 | 5 (2) | 22 | 66.7 |

^a^R denotes a **revised** indicator; N denotes a **new** indicator suggested by the panel

# S4 Table 6. Round three survey indicators that achieved ≥ 70% consensus level on both criteria

|  | **Important** | | | | **Actionable** | | | |
| --- | --- | --- | --- | --- | --- | --- | --- | --- |
| **Indicators (n=3)**^a^ | **Median (IQR)** | **No.** | **Consensus Level (%)** | **n** | **Median (IQR)** | **No.** | **Consensus Level (%)** | **n** |
| **Governance and Leadership** |  |  |  |  |  |  |  |  |
| 2N. The public health agencies plans are linked to the mandate of network partners in vertical or horizontal multi-jurisdictional response to emergencies; for example, responsibilities for different levels of government. | 5 (0) | 23 | 88.5 | 26 | 5 (1) | 23 | 88.5 | 26 |
| 4N. The public health agency engages with policy-makers to address gaps in policy and/or legislation that pertain to the effectiveness of its emergency management plans and/or protocols. | 5 (1) | 24 | 92.3 | 26 | 5 (1) | 22 | 91.7 | 24 |
| **Resources** |  |  |  |  |  |  |  |  |
| 7N. The public health agency has mechanisms to secure or reallocate financial resources to support response to and recovery from an emergency. | 5 (1) | 26 | 96.3 | 27 | 4 (1) | 20 | 80 | 25 |

^a^R denotes a **revised** indicator; N denotes a **new** indicator suggested by the panel

n = number of panel members who voted in indicator poll

# S4 Table 7. Discarded indicators due to low consensus on agreement for importance and actionability (< 70%)

|  | Important | | | | Actionable | | | |
| --- | --- | --- | --- | --- | --- | --- | --- | --- |
| Indicators (n=2)^a^ | Median  (IQR) | No. | Consensus Level (%) | n | Median (IQR) | No. | Consensus Level (%) | n |
| Collaborative Networks |  |  |  |  |  |  |  |  |
| 28. The public health agency has established processes, in cooperation with network partners, to effectively manage differences and disputes in the context of an emergency. | 3 (3) | 12 | 48 | 25 | 2 (1.5) | 6 | 25 | 24 |
| Learning and Evaluation |  |  |  |  |  |  |  |  |
| 56. The public health agency has defined an evaluation, learning and/or research agenda that aims to address knowledge gaps relevant to emergency management. | 4 (3) | 15 | 57.7 | 26 | 4 (2.75) | 16 | 61.5 | 26 |

^a^n = number of panel members who voted in indicator poll

# S4 Table 8. Important (≥ 70%) but not actionable (≤ 70%) indicators

|  | **Important** | | | | **Actionable** | | | |
| --- | --- | --- | --- | --- | --- | --- | --- | --- |
| **Indicators (n=7)**^a^ | **Median (IQR)** | **No.** | **Consensus**  **Level (%)** | **n** | **Median (IQR)** | **No.** | **Consensus**  **Level (%)** | **n** |
| **Governance and Leadership** |  |  |  |  |  |  |  |  |
| 5N. Provincial/territorial authorities and local/regional public health agencies jointly develop policies and/or structures defining the agency mandate in public health emergency management. | 5 (1) | 24 | 88.9 | 27 | 3.5 (3.75) | 13 | 50 | 26 |
| **Collaborative Networks** |  |  |  |  |  |  |  |  |
| 8N. The public health agency and its network partners have demonstrated shared understanding of roles, responsibilities and procedures relevant to the emergency context. This understanding may be demonstrated, for instance, during real or simulated emergencies. Network partners may include partners within or outside the health sector and vertical or horizontal partners, based on the agency context. | 5 (1) | 23 | 88.5 | 26 | 4 (3) | 16 | 61.5 | 26 |
| 9N. The public health agency has mutual aid agreements in place with network partners outside the health sector that describe how resources and/or services will be shared during an emergency, including meeting demands for surge capacity; for example, non-governmental organizations such as the Red Cross. | 5 (1) | 24 | 92.3 | 26 | 2 (3) | 10 | 38.5 | 26 |
| **Community Engagement** |  |  |  |  |  |  |  |  |
| 32. The public health agency has a process to ensure transparency with the public with respect to what is known, what is not known and where there are genuine differences of professional opinion; for example, in the context of uncertain science or differences with Indigenous knowledge. | 5 (2.5) | 17 | 73.9 | 23 | 2.5 (3) | 11 | 45.8 | 24 |
| **Workforce Capacity** |  |  |  |  |  |  |  |  |
| 47. If the use of citizen volunteers is permitted by the public health agency to support its mandate in emergencies, the agency has ensured there is a process for engaging volunteers. This may include volunteer recruitment, identification, credentialing, and/or training linked to processes in the broader health or government services sector. | 5 (1) | 21 | 84 | 25 | 4 (2.75) | 15 | 68.2 | 22 |
| **Surveillance and Monitoring** |  |  |  |  |  |  |  |  |
| 52. Local/regional laboratories (e.g., commercial and/or hospital) have the capability for timely reporting of results to the public health agency for surveillance relevant to public health emergencies. | 6 (0) | 25 | 100 | 25 | 4 (2.5) | 17 | 63 | 27 |
| **Learning and Evaluation** |  |  |  |  |  |  |  |  |
| 57. The public health agency has resources allocated and/or identified for evaluation, learning and/or research to address knowledge gaps relevant to emergency management. | 5 (1) | 22 | 88 | 25 | 3 (3) | 12 | 48 | 25 |

^a^R denotes a **revised** indicator; N denotes a **new** indicator suggested by the panel

n= the number of participants who voted in each poll

# S4 Table 9. Summary of decisions made by the panel in round 3

| **Round 3 Indicator Proposed** | **Comments from Panel** | **Outcome** |
| --- | --- | --- |
| 28. The public health agency has established processes to effectively manage conflicts with network partners in the context of an emergency. | - This is relative to the IMS. There is a crisis management structure that is already in place so there may not be a need for a formal process. - This indicator may not be relevant to all the different types of public health agencies. | The panel confirmed that the indicator should be discarded from the final list of PHEP indicators. |
| 32. The public health agency strives for transparency with the public where there are genuine differences of professional opinion; for example, in the context of uncertain science or differences with Indigenous knowledge. | - There are difficulties in controlling differences of opinion with experts and professionals outside of the agency. - The indicator is not distinct from risk communication. - Good communication is excellent in principle, but it may not be the local public health agency’s responsibility. - The indicator would be tough to action. | The indicator was found to be important but agreement on actionability was low. The indicator will not be included in the final indicator set. Further research and adaptation will be required. |
| 47. If the use of citizen volunteers is permitted by the public health agency to support its mandate in emergencies, the agency has a process for integrating volunteers. This may include volunteer recruitment, identification, credentialing and/or training linked to processes in the broader health-sector. | - Integrating volunteers may not be the responsibility of the public health agency. - Based on the discussion of panel members, the indicator was re-worded to state:   *If the use of citizen volunteers is permitted by the public health agency to support its mandate in emergencies, the agency has ensured there is a process for engaging volunteers. This may include volunteer recruitment, identification, credentialing, and/or training linked to processes in the broader health or government services sector.* | The rephrased indicator reached consensus of agreement for importance and nearly approached the threshold of consensus for actionability. The indicator will not be included in the final indicator set. Further research regarding a process for integrating volunteers will be required. |
| 56. The public health agency has defined a research or evaluation agenda that aims to address knowledge gaps relevant to emergency management. | - Although this indicator is specific to addressing knowledge gaps, the broad focus of research and evaluation for process improvement has already been captured in three other indicators. | The panel confirmed that the indicator should be discarded from the final set of PHEP indicators. |
| 57. The public health agency has resources allocated and/or identified for evaluation or learning to address relevant knowledge gaps for the agency in emergency management. | - Similar to indicator 56, the broad focus of research and evaluation for process improvement has already been captured in three other indicators. | The indicator was found to be important but did not reach the threshold for consensus of agreement for actionability. The indicator will not be included in the final indicator set. Further research and adaptation will be required. |
| 2N. The public health agency’s policies describe the mandate of network partners in vertical or horizontal multi-jurisdictional response to emergencies; for example, responsibilities for different levels of government. | - Whether or not describing the mandate of network partners should be a public health agency's role versus the broader emergency management agency within the province was the focus of discussion. - A suggestion was made to change the wording from policies to *plans*. - The indicator was revised to state:   *The public health agencies plans are linked to the mandate of network partners in vertical or horizontal multi-jurisdictional response to emergencies; for example, responsibilities for different levels of government.* | The revised indicator reached consensus of agreement for both importance and actionability. The indicator will be added to the final set of PHEP indicators. |
| 4N. The public health agency engages with policy-makers to address gaps in policy and/or legislation that pertain to the effectiveness of its emergency management plans and/or protocols. | - Discussion ensued regarding the meaning of the term *engagement*. Is it a matter of enabling change? Is it about raising an issue? - The panel agreed that in principle, the indicator is actionable. The indicator requires the public health agency to engage or communicate with policy makers to address gaps. | The indicator reached consensus of agreement for both importance and actionability. The indicator will be added to the final set of PHEP indicators. |
| 5N. Provincial/territorial authorities and local/regional public health agencies jointly develop policies and/or structures defining the agency mandate in public health emergency management. | - Policy development regarding mandates for public health emergency management is the responsibility of the provincial/territorial authorities, not the responsibility of local/regional public health agencies. | The indicator reach consensus for agreement of importance but not actionability. The indicator will not be included in the final indicator set. This indicator will continue to be reviewed in future research. |
| 7N. The public health agency has mechanisms to secure or reallocate financial resources to support response to and recovery from an emergency. | - There is a challenge in reallocating financial resources within the government. - The mechanism is fundamentally provincial/territorial, or at times, a federal responsibility. - A health agency does not have control over its own finance mechanisms. The mechanisms are in the control of external entities. - There are accountabilities and explanations that have to be made in long-term requests for provincial monies; however, in the short-term, there are opportunities to reallocate finances. | The indicator reached consensus of agreement for both importance and actionability. The indicator will be added to the final set of PHEP indicators. |
| 8N. The public health agency and its network partners have demonstrated shared understanding of roles, responsibilities and procedures relevant to the emergency context. This understanding may be demonstrated, for instance, during real or simulated emergencies. Network partners may include partners within or outside the health sector and vertical or horizontal partners, based on the agency context. | - The dependency on others to demonstrate an understanding is impacting the criteria of actionability. What a network partner can demonstrate is out of the control of a public health agency. | This indicator was deemed to be important but did not reach the necessary level of consensus of agreement for actionability. Future research will consider the interdependency of network relationships and how this may impact joint undertakings in PHEP activities. The indicator will not be included in the final indicator set. |
| 9N. Mutual aid agreements are in place between the public health agency and network partners outside the health sector that describe how resources and/or services will be shared during an emergency, including meeting demands for surge capacity. For example, non-governmental organizations such as the Red Cross. | - Ensuring that there is access to a mutual aid agreement is important, but it is not necessarily between the agency and its partners. A mutual aid agreement may be between the municipality and the public health agency, or between the agency and another entity that the agency is part of. - It's really important for the system, but it's not actionable at the local level. - The indicator speaks to the difficulty in disentangling the responsibilities and the role of a public health agency within a very interdependent system. | The indicator obtained the level of consensus for agreement of importance but was deemed to not be actionable. Future research will explore the function of mutual aid agreements regarding sharing of resources/services and which jurisdictional level this responsibility pertains to. The indicator will not be included in the final indicator set. |
| 52. Local/regional laboratories (e.g., commercial and/or hospital) have the capability for timely reporting of results to the public health agency for surveillance relevant to public health emergencies. | - The legislative framework for surveillance reporting and privacy is at the authority level of the province. - There is potential to develop a separate indicator around the timeliness of reporting by labs. | The panel was unanimous that the indicator was important; however, not all members agreed that the indicator was actionable at the local/regional level. Future research will focus on timely reporting by local/regional laboratories. The indicator will not be included in the final indicator set. |
